# Memristive Ion Dynamics to Enable Biorealistic Computing

**DOI:** 10.1021/acs.chemrev.4c00587

**Published:** 2024-12-27

**Authors:** Ruoyu Zhao, Seung Ju Kim, Yichun Xu, Jian Zhao, Tong Wang, Rivu Midya, Sabyasachi Ganguli, Ajit K. Roy, Madan Dubey, R. Stanley Williams, J. Joshua Yang

**Affiliations:** † Ming Hsieh Department of Electrical and Computer Engineering, University of Southern California, Los Angeles, California 90089, United States; ‡ Sandia National Laboratories, Livermore, California 94550, United States; § Air Force Research Laboratory Materials and Manufacturing Directorate Wright − Patterson Air Force Base Dayton, Ohio 45433, United States; ∥ Sensors and Electron Devices Directorate, U.S. Army Research Laboratory, Adelphi, Maryland 20723, United States; 1 Department of Electrical & Computer Engineering, Texas A&M University, College Station, Texas, 77843, United States

## Abstract

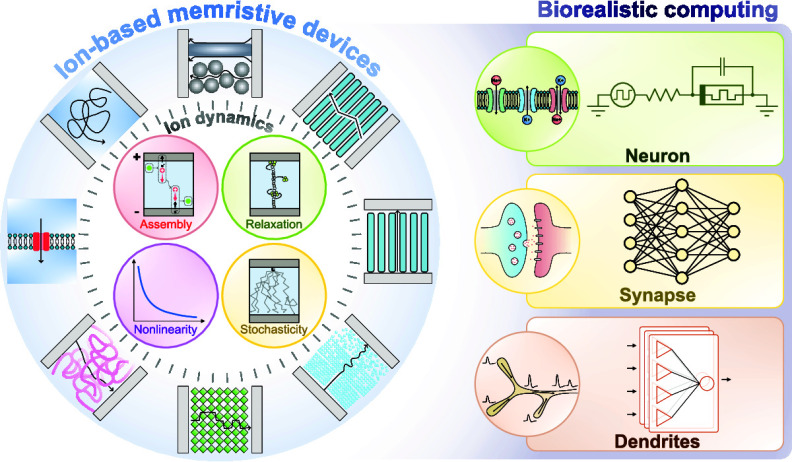

Conventional artificial intelligence (AI) systems are facing bottlenecks due to the fundamental mismatches between AI models, which rely on parallel, in-memory, and dynamic computation, and traditional transistors, which have been designed and optimized for sequential logic operations. This calls for the development of novel computing units beyond transistors. Inspired by the high efficiency and adaptability of biological neural networks, computing systems mimicking the capabilities of biological structures are gaining more attention. Ion-based memristive devices (IMDs), owing to the intrinsic functional similarities to their biological counterparts, hold significant promise for implementing emerging neuromorphic learning and computing algorithms. In this article, we review the fundamental mechanisms of IMDs based on ion drift and diffusion to elucidate the origins of their diverse dynamics. We then examine how these mechanisms operate within different materials to enable IMDs with various types of switching behaviors, leading to a wide range of applications, from emulating biological components to realizing specialized computing requirements. Furthermore, we explore the potential for IMDs to be modified and tuned to achieve customized dynamics, which positions them as one of the most promising hardware candidates for executing bioinspired algorithms with unique specifications. Finally, we identify the challenges currently facing IMDs that hinder their widespread usage and highlight emerging research directions that could significantly benefit from incorporating IMDs.

## 1 Introduction

Artificial intelligence (AI) has experienced unprecedented growth in the past decade. The latest AI models demonstrate their remarkable capabilities across various domains, including conversational agents (ChatGPT), gaming (AlphaGo Zero), research (AlphaFold), and art (Sora, Suno, and Midjourney), even surpassing human performance in some aspects.^1−6^ However, these impressive achievements come at a significant cost in terms of money, energy, and time during the training process. The demand for computing resources to train state-of-the-art AI models is doubling every two months, a rate that far outpaces the historical rate of Moore’s law.^7^ This unsustainable trend necessitates the exploration of more efficient devices, systems, and algorithms for future AI. Biological systems, in contrast, consume significantly less energy and occupy much less space than current AI systems, while exhibiting more comprehensive learning capabilities and more robust operational performance. Inspired by this efficiency, researchers have been striving to emulate the behaviors of biological neural networks (BNNs) to develop bioinspired computing systems that are both efficient and powerful.^8,9^

BNNs exhibit complex dynamics originating from the transport of discrete units, such as the flow of electrolyte ions and neurotransmitters, which introduce stochastic variations and are believed to be the key to the effectiveness of BNNs.^10,11^ Traditional computing cores, such as central processing units (CPUs) and graphics processing units (GPUs), are designed for deterministic digital computing. They are very inefficient when implementing bioinspired algorithms like spiking neural networks (SNNs). To address this, customized complementary metal-oxide-semiconductor (CMOS) circuits have been developed to emulate biological behaviors.^12−14^ However, due to the intrinsic differences in operation mechanisms of transistors and neurons, these circuits require complex designs with substantial numbers of components, especially large capacitors, which limit the optimization of their size and power.

Memristive devices, with the dynamic relationships between their conductance and the stimulus applied to them, are promising for implementing biorealistic dynamics at the single-device level.^15−18^ Various mechanisms can induce memristive behaviors, including ion transport, phase change, magnetic polarization, and ferroelectric polarization.^19^ Among these, phase change, ferroelectric switching, and magnetization switching typically involve reconfigurations of atomic lattices or electron spins on lattices between specific arrangements without any long-range motion, limiting their capabilities to exhibit complex or continuous dynamics. In contrast, ion transport stands out as it typically involves stochastic long-range motion, leading to complex dynamic processes that are intrinsically similar to those in biological neurons.^20−22^ Consequently, memristive devices based on ion migration, referred to as ion-based memristive devices (IMDs) in this paper, are superior candidates for realizing highly desirable biorealistic dynamics. IMDs are built using various active ions, switching materials, and structural configurations, all of which influence their switching behaviors. Understanding the fundamental mechanisms behind these dynamic behaviors and their relation to device materials and structures is crucial for developing IMDs with desired properties.

This review begins with an analysis of the dynamics within IMDs in Section 2, exploring the fundamental components of the diverse dynamic behaviors and the potential mechanisms involved. In Section 3, we classify IMDs based on their switching material types and discuss how different switching materials can lead to distinct dynamics. Section 4 surveys the applications of IMDs in creating essential components of biorealistic computing systems and compares the performances of IMD-based artificial components with their biological counterparts, highlighting their potential and challenges. Section 5 discusses various methods to tune and optimize the IMDs, providing comprehensive guidance for developing more biorealistic devices. Finally, in Section 6, we address the major challenges for the general applications of IMDs and propose four emerging research directions involving IMDs that are critical for advancing bioinspired artificial intelligence.

## 2 Mechanism of Ion-Based Memristive Devices

### 2.1 Dynamics

**Figure 1:**
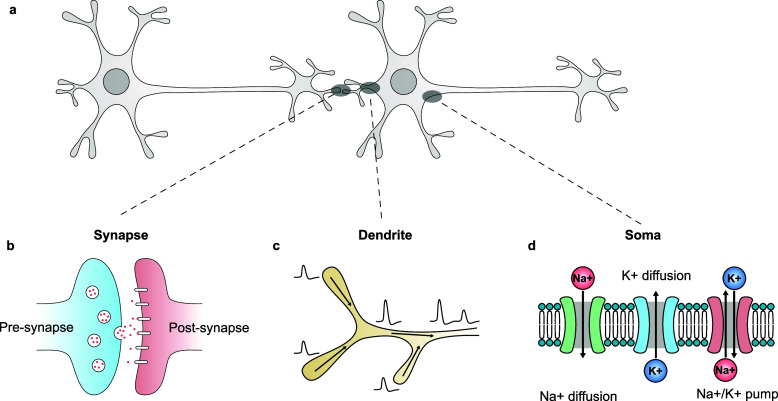
Fundaments of biological neural dynamics.^11,23,24^ (a) Dynamic behaviors in biological neural systems are based on the information transmission from one neuron to another via the synapses and the dynamic processes within the dendrites and soma of the neuron. (b) In the synapses, neurotransmitters are released from the presynaptic neuron and combine with the receptors on the postsynaptic neuron under dynamic migration. (c) In the dendrites, ion concentrations along the dendrites are changing dynamically due to the propagation of action potentials from various directions. (d) In the somas and other parts of the biological neuron, ions between the biological membrane are dynamically regulated by active ion pumps and passive ion channels.

The rich dynamic behaviors within the BNNs are the fundaments of their highly efficient and powerful learning performance (Figure 1). Though these behaviors are various, they all originate from a few basic processes involving the transport of units from site to site (ions, transmitters, etc.), such as the accumulation of particles driven by ATP consumption and the diffusion of the particles in a concentration gradient, the nonlinear dynamics responding to these particles, such as the opening and closing of ion channels and transmitter acceptors, and the stochasticity involved during these discrete processes.^11,23,24^ There are good counterparts of all these processes within the IMDs since they also function based on ion or particle movement. In this section, we describe how these corresponding processes occur within the IMDs.

#### 2.1.1 Assembly

The ON-switching process, i.e. increased electrical conductivity, of the IMDs generally involves the accumulation of active ions under an external stimulus. Here we refer to this as the assembly process.

**Figure 2:**
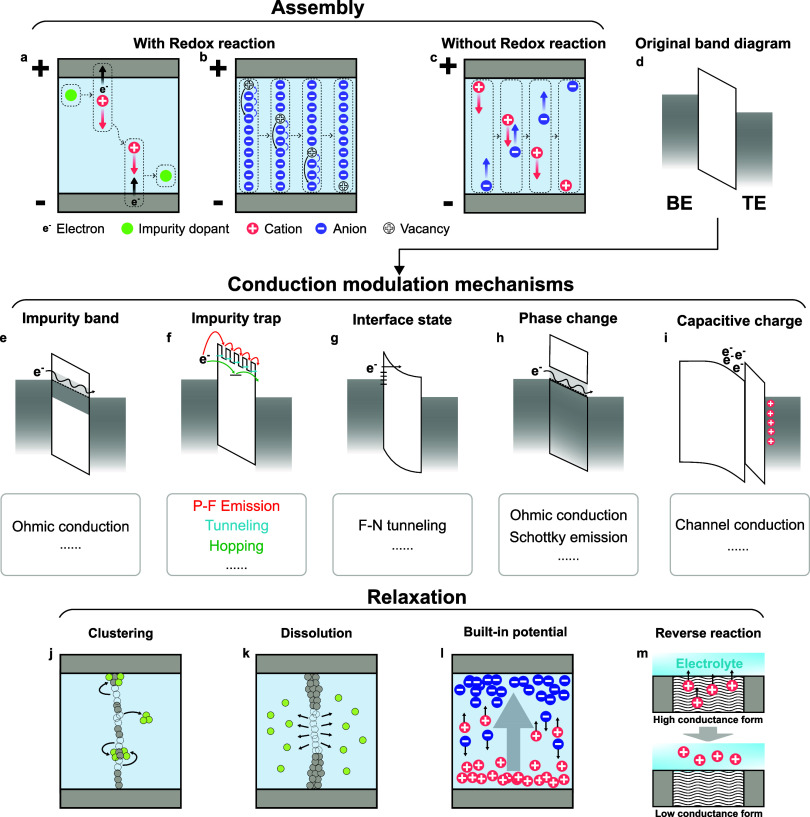
Switching dynamics of the IMDs. The assembly processes of the ions are driven by external electrical stimulation. Dopants such as (a) impurity atoms and (b) vacancies undergo redox reactions during structure assembly.^19^ (a) Impurity atoms, such as Ag, transform to charged ions, migrate under the external bias, and then transform back to the neutral status. (b) Vacancies, such as oxygen vacancies in metal oxides, exhibit migration behavior due to the movement of the oxygen anions, which involves valence changes of the corresponding metal ions (not shown here) in the metal oxides. The simultaneous and collective movement of multiple anions results in an equivalent movement of an oxygen vacancy in the opposite direction, with an effectively higher speed. For (c) native ions, they are originally charged and are moved under external bias directly without redox processes.^27−30^ The assembly processes result in the devices changing from (d) the original insulating into conductive status with different types of conduction channels enabled by ion migration, including (e) impurity bands,^35^ (f) impurity traps,^36^ (g) interface states,^37,38^ (h) conductive phases,^39,40^ and (i) double-layer charges.^27,28^ The most common conduction mechanisms are listed underneath the corresponding band diagrams. The accumulated ions during the assembly processes relax spontaneously via different mechanisms, including (j) clustering,^25^ (k) dissolution,^39,43^ (l) built-in potentials,^29,44^ and (m) reversible chemical reactions,^40,45^ when there are no external stimuli.

There are many ways to realize the transport and accumulation of the ions. The most common within the IMDs are shown in Figure 2a to 2c. If the active atoms (or vacancies) of the memristive devices tend to stay neutral within the switching materials, such as Ag, Cu, oxygen, or oxygen vacancies,^19,25^ these particles first undergo ionizing processes under the influence of an external electric field, to transform into cations or anions (Figure 2a and 2b). The ions then drift under electric fields toward the opposite side of the device and transform back into neutral species once they exchange electrons with the electrode. In this case, the ion mobility and the redox reaction rate interact to influence the assembly process.^26^ If the active ionic species remain charged during the entire switching process, such as electrolyte ions^27,28^ or halide perovskite ions,^29,30^ the ions drift under electric fields without any redox reaction (Figure 2c). Other than electric fields, external stimuli such as light,^31^ heat,^32^ or chemical species^33,34^ can also influence the transport of ions.

**Figure 3:**
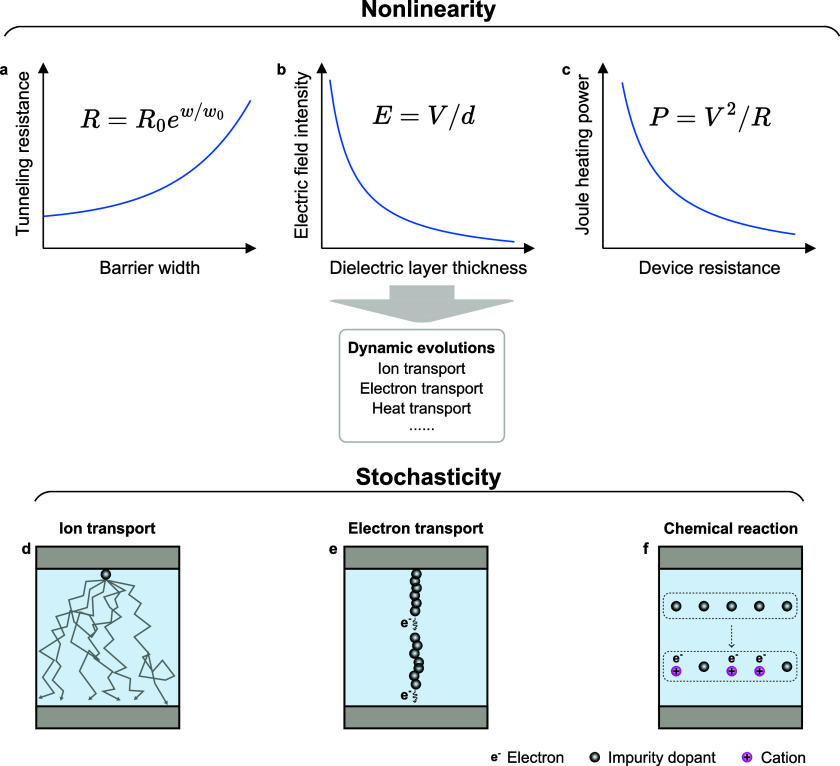
Different nonlinear relationships within the dynamic evolutions, such as (a) tunneling resistance versus tunneling barrier width,^36^ (b) electric field intensity versus dielectric layer thickness,^58^ and (c) Joule heating power versus device resistance,^41,51−53^ and various sources of stochasticity within (d) ion transport, (e) electron transport, (f) chemical reactions, etc., are involved in the dynamic processes within the IMDs, making the device operating as a stochastic nonlinear dynamic system.^10,46^

**Figure 4:**
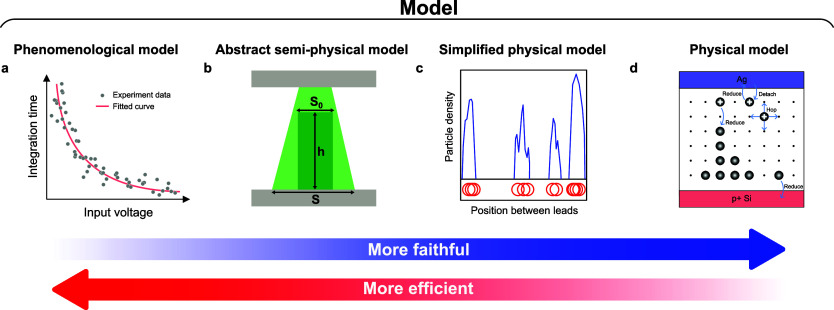
IMDs can be modeled at different levels. (a) Phenomenological models use fitted curves from measurement results to describe the device behaviors.^59^ (b) Abstract semiphysical models ignore the details of IMDs and use a few variables, such as the filament height h and filament area S, to capture the device status and evolution.^50,65^ (c) Simplified physical models include the detailed positions and movements of all the particles inside the IMDs but limit their movement to 1D or ignore some trivial influences.^41,64^ (d) A complete physical model captures the detailed movements of all the ions.^60,61^ (c) Reproduced with permission from ref (41). Copyright 2017, Springer Nature. (d) Reproduced with permission from ref (61). Copyright 2019, Wiley-VCH.

**Figure 5:**
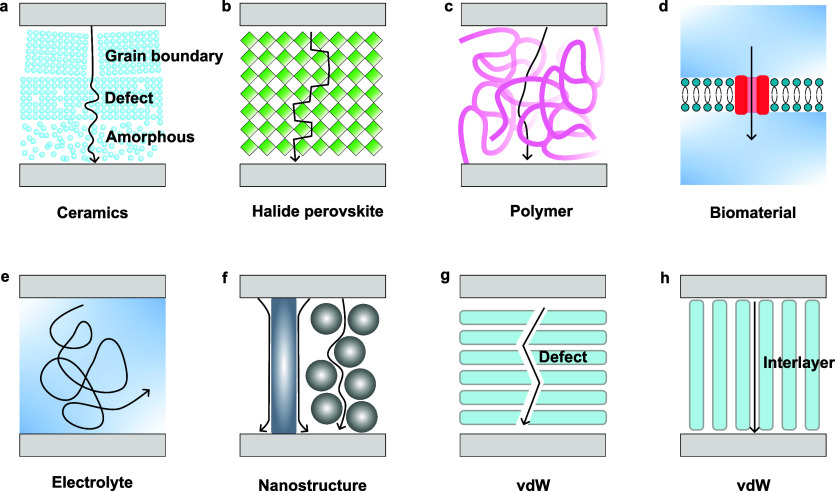
IMDs are formed with various switching materials. Different types of switching materials provide different types of ion migration paths. (a) Ceramic switching materials utilize their grain boundary, localized defects, and amorphous structures as the ion migration paths.^25,39,41,42,69^ (b) Halide perovskites allow ions to move within interstitial sites inside their crystal structures.^70^ (c) Polymers provide the interfaces of the polymer molecules as the ion migration paths.^71^ (d) Biomaterials can directly form ion channels or other biological structures as the ion migration paths.^72,73^ (e) Electrolytes allow ions to move freely.^74,75^ (f) Nanostructures provide surfaces as the ion migration paths.^76−78^ VdW materials can use (g) defects and grain boundaries, or (h) interlayer spaces to transport ions.^79^

The migration of ions during assembly processes leads to a conductance change, i.e. ON-switching, of the memristive devices. Figure 2e to 2i show the common mechanisms of conductance change due to ion migration within IMDs: 1) the ions form a continuous conductive filament within the switching materials and introduce an impurity conductive band connecting the two electrodes for electron migration^35^ (Figure 2e); 2) the ions form many separated impurity traps within the switching materials so that the electrons can migrate between the two electrodes with the help of these traps based on Poole-Frankel emission, tunneling, or hopping effects^36^ (Figure 2f); 3) the ions accumulate on the interface of electrodes and switching materials, increase the slope of the Schottky barrier, and result in Fowler-Nordheim tunneling^37,38^ (Figure 2g); 4) the ions interact with the switching materials and change the switching materials from insulating phases, with large band gaps, to conductive phases, with small band gaps^39,40^ (Figure 2h); 5) for three-terminal or transistor-type memristive devices, the ions accumulate at the interface between switching layers and the transistor channel, leading to the accumulation of carriers (holes or electrons) within the channels^27,28^ (Figure 2i).

#### 2.1.2 Relaxation

After ON-switching, the conductivity of the IMDs usually decreases with time spontaneously when there is no external stimulus, which is referred to as the relaxation process within this review. Four commonly observed mechanisms lead to the relaxation process, as shown in Figure 2j to 2m. The first type is driven by interfacial energy minimization, or Ostwald ripening, which leads to the dissolution of the atoms in filaments and reformation into spheres or absorption onto electrodes (Figure 2j). This is usually observed in devices based on metal ion migration, such as Ag and Cu.^25^ Since these metal atoms have low migration barriers and high interfacial energies with the switching materials, they tend to aggregate together to reduce their interfacial energy under thermal movement, which in general ruptures the thinnest parts of the metal filaments. The rupture and cluster of metal filaments are observed in situ during switching.^41^ Since this process is driven by both the diffusion and the absorption of the active ions, it can be very fast, reaching a nanosecond-level response time.^42^ The second type of relaxation process is driven by the dissolution of the conduction channel under ion concentration gradients (Figure 2k). Within the memristive devices based on ions that do not preferentially congregate, such as oxygen vacancies and protons,^39,43^ or the metal filament is thick enough to attain low interfacial energy, the conduction channels tend to reduce with the dissolution of ions from the higher-concentration region to the lower-concentration region. Since this process relies purely on the random diffusion of the ions, it usually takes much longer time than the first type, reaching one second or even longer. Besides these two types of relaxation mechanisms, the movement of active ions may also be driven by the built-in potentials within the switching layer (Figure 2l), which could be a result of the Fermi level difference between the two electrodes of the memristive device^44^ or the accumulation of opposite ion charges on the two sides of the switching materials.^29^ In this case, the relaxation speed is correlated to the built-in field intensity. Lastly, the relaxation process can also result from a reversible electrochemical reaction (Figure 2m). For memristive devices that change their conductance under electrochemical reactions of the switching material, the reaction may be reversed when there is no external driving force, such as the behaviors observed in memristive transistors using the organic ionic-electronic conductors as the channel materials.^40,45^

#### 2.1.3 Nonlinearity

An important feature that leads to the rich neuromorphic applications of IMDs is their highly nonlinear switching,^46^ making the entire circuit a nonlinear dynamical system. Here, we present three types of commonly encountered nonlinearities in the memristive devices: the relationship of the tunneling conductance versus the tunneling gap width, the electric field intensity versus the dielectric layer thickness, and the joule heating power versus the device resistance. These represent the nonlinearities induced by quantum effects, electromagnetism, and thermal dynamics (Figure 3a to 3c). There are nonlinear dependencies on physical variables for many conduction mechanisms (Figure 3a), such as the electron emission through a Schottky barrier,^47^ and the trap-assisted tunneling from defect to defect.^48^ Additionally, the electric field intensity has a highly nonlinear dependence on both dielectric layer thickness and conductive electrode sharpness (Figure 3b). For devices with a conduction filament growing under an electric field, the electric field intensity increases rapidly at the tip of the filament, resulting in positive feedback during the growth process and swift switching behavior.^49,50^ Moreover, the temperature variations caused by Joule heating and the subsequent thermal conduction (Newton) cooling strongly change the electrical conductivity (Figure 3c), which is an important factor leading to high-order memristive behaviors.^41,51−53^ With all these factors playing together, the switching process of IMDs is highly nonlinear and diverse, realizing nonlinear selectivity,^42^ nonlinear resistance,^54^ nonlinear memory decay,^55^ etc., which can be used to emulate the nonlinearity and complexity within biological neural systems. Biological neural networks are proven to be high-dimensional nonlinear systems, with the biological neurons observed to operate with nonlinear dynamics.^56^ The nonlinearity is considered an important origin of the great computational capability of biological neural systems. Thus, in order to acquire the same computational potential, biorealistic computing also seeks to use nonlinear components and build nonlinear dynamic systems.^46^

#### 2.1.4 Stochasticity

Noise and stochasticity, which are highly undesirable in digital computing systems, are ubiquitous within the operation of IMDs. For neuromorphic applications, stochasticity enriches the complexity of the device’s dynamic switching and makes the device more biorealistic.^57^ There are three typical sources of stochasticity within the IMDs: ion transport, electron transport, and chemical reactions (Figure 3d to 3f). Ions randomly diffuse with and without an external bias (Figure 3d). This significantly affects the locations and shapes of a conduction channel, leading to cycle-to-cycle (C2C) variability of the IMDs. Multiple types of noise are involved during electron transport (Figure 3e), such as thermal noise, shot noise, flicker noise, and quantum noise, which influence the output currents through the IMDs and contribute to the dynamic evolution that can produce large behavioral variations. The ion migration within IMDs is also affected by the randomness of chemical reactions (Figure 3f), such as redox and intercalation/deintercalation, influencing the assembly of conduction channels.

### 2.2 Modeling

Compact modeling of IMDs holds significant promise in enhancing our understanding of device behavior, enabling accurate property predictions and comparisons across diverse materials, and facilitating efficient circuit simulations with such devices. Additionally, it provides a foundation for algorithm development based on the rich dynamic behaviors of the devices and paves the way toward large-scale intelligent systems involving memristive devices.

Currently, various models at different levels of abstraction have been proposed to capture the intricate dynamics of these devices.^58^ Some models describe device behaviors using theoretical or empirical equations to correlate current and response time to other physical parameters such as voltage, temperature, and device area, and then fit these equations to experimentally measured data^59^ (Figure 4a). These models ignore the details of active ion migration and conduction channel morphology, and thus, do not fully depict the dynamic processes. On the contrary, some physical models consider the detailed behavior of all the active ions inside the memristive devices (Figure 4d). Kinetic Monte Carlo (kMC) is used to simulate the migration of all the individual active ions inside the switching layer, as well as the redox reactions involved.^60,61^ Finite element simulations model the evolution of filament morphology, thermal dissipation, and electric field distribution from a mesoscopic view.^51,62,63^ These simulation techniques are time-consuming due to the heavy computational load and unsuitable for circuit modeling. To improve the computational efficiency, researchers have utilized a simplified 1D model and abstracted the ion distributions as a string of nanoparticles (Figure 4c). These abridged physical models can describe the electrical, chemical, and thermal^64^ influences on each nanoparticle, but still require significant computational resources if they are used to simulate multiple devices within larger systems.

To achieve circuit or system-level simulations with IMDs, recent efforts have been focused on developing compact and computationally efficient models that preserve the essential dynamic features of the devices, while simplifying the details as much as possible (Figure 4b). Instead of tracking individual ions or nanoparticles, the models abstract a few necessary state variables (such as filament length and width) and use dynamical differential equations to describe their behavior under bias conditions.^50,65^ These models are suitable for simulating circuits and networks that are composed of memristive devices. Nonetheless, other variables are needed to describe the device behavior more accurately, such as the ion residue within the switching layer and stochastic behavior,^66,67^ to make the model more generalized for various applications. Recently, a comprehensive model has been presented to simulate the behaviors of diffusive memristors, incorporating the dynamics of filament growth and decay, ion residue, and stochasticity.^68^ This model faithfully replicates both the static and dynamic behaviors of the diffusive memristor, while maintaining minimal computational requirements, opening the avenue for large-scale circuit simulation and circuit/algorithm co-design.

## 3 Types of Ion-Based Memristive Devices

There are different ways to classify IMDs, such as classification based on their active ions, switching mechanisms, or conduction mechanisms. In this review, we classify these devices based on the material types of their switching layers, which determine the morphology and properties of ion migration paths and constrain the ion movements.

### 3.1 Ceramics

Most ionic memristive devices are based on ceramic switching layers, such as oxides, nitrides, and carbides. These materials usually possess amorphous or polycrystalline structures, with rich defects or grain boundaries that can serve as the ion migration paths (Figure 5a). Under an electric field, the ions drift to form conduction channels along these migration paths. Since the ions are not stable inside ceramic materials, they usually stay neutral within the material and only transform to ionic states after redox reaction under external bias.

Ag is the most used cation for ceramic-based memristive devices, due to its low energy barrier for redox reactions and small atom size for moving inside switching materials. Ag ions exhibit dynamic or volatile switching behaviors in HfO_*x*_,^41,42^ SiO_*x*_,^67,80^ TiO_*x*_,^81,82^ SiO_*x*_N_*y*_,^41^ MgO_*x*_,^41,83^ ZnO_*x*_,^84,85^ TaO_*x*_,^86,87^ AlO_*x*_,^88^ and ZrO_*x*_.^89^ Cu is often used as an active cation as well, with a higher redox reaction barrier and lower mobility compared to Ag. Cu is CMOS compatible and used for interconnects within the back-end-of-line (BEOL) process, so memristive devices using Cu as contact electrodes and mobile ions may be readily compatible with the CMOS process. Cu is demonstrated to achieve volatile or dynamic switching behaviors in various ceramic materials, such as HfO_*x*_,^90,91^ AlO_*x*_,^92^ SiO_*x*_,^93^ TiO_*x*_,^94^ and SiC.^95,96^ Besides Ag and Cu, other cations are also demonstrated to show dynamic switching behavior in ceramic switching layers, such as carbon in Ga_2_O_3_,^97^ Mg in MgO,^98^ and Te in Ta_2_O_5_.^99^ The active ions inside the ceramics can also be anions. TiO_*x*_ is the most used material with active oxygen anions,^39,100,101^ and some researchers also believe both titanium cations and oxygen anions are involved in the switching process.^102^ Other ceramic materials are also used to demonstrate switching behavior based on oxygen anion migration, such as SiO_*x*_,^103^ TaO_*x*_,^51^ HfO_*x*_,^104^ SnO_*x*_,^105^ and ZnO.^106^

Due to the high resistivity of ceramics, this type of memristive device can be very thin while maintaining a high ON/OFF ratio. Furthermore, due to the high mobility of active ions within grain boundaries or defects, the switching voltage can be very low, and the switching speed can be very fast, leading to an efficient and fast switching process. However, the defect distribution or grain boundary distribution within ceramic materials is random and difficult to control during the fabrication process, which increases the device-to-device (D2D) variations. The complex distribution of migration paths also randomizes the filament growth from cycle to cycle (C2C). Moreover, the large variation may result in thick and nonvolatile filaments, leading to device failure and poor endurance.

### 3.2 Halide Perovskites

Halide perovskites (HPs) are emerging materials in optoelectronics because of their outstanding physical properties, such as high mobility, majority carrier control, long carrier lifetime, and tunable bandgap. Therefore, they have been actively studied for optoelectronic applications such as photovoltaics (PV), light detectors (including photo, X-ray, and γ-ray, light-emitting diodes (LEDs)), and lasers. The typical crystal structure of 3D HPs is ABX_3_ with six-coordinated BX_6_ octahedra connected by a corner-sharing structure. A is an organic or inorganic cation, typically methylammonium (MA^+^), formamidinium (FA^+^), or cesium ion (Cs^+^); B is Pb^2+^, Sn^2+^, or Ge^2+^; X is I^–^, Br^–^, or Cl^–^. This unique crystal lattice enables wide valence and conduction bands, and the favorable electronic structure facilitates charge transport.

HPs have been proposed as the switching layer materials for memristive devices.^70,107,108^ The soft lattice matrix of HPs has a low activation energy for ionic transport and enables fast and controllable ion movement across the whole HP layer (Figure 5b). Compared to other dielectric materials, such as metal oxides or metal sulfides, the soft lattice matrix of HP materials has lower activation energy for Ag migration (i.e., ZrO_2_ is 2.48 eV,^109^ Ag_2_S is 0.67 eV,^110^ and MAPbI_3_ is 0.25 eV^111^). Therefore, the ions in HPs tend to have higher mobility, enabling fast and controllable ion movement across the whole HP layer and low operation voltages for high energy efficiency. In addition, the resistive switching behavior of halide perovskites shows rich possibilities due to the diverse ion transport physics including interfacial doping, electrochemical metallization with electrodes, controlled ion migration with mixed-ionic-electronic conductivity, and the diffusive dynamics of metal conductive filaments. Very recently, low-dimensional HPs such as HP quantum dots, HP nanorods, two-dimensional, and quasi-two-dimensional HPs are being explored to build memristive devices with rich dynamics and show great promise.^112,113^

The native ions within HP materials, such as MA^+^, FA^+^, Cs^+^, I^–^, and Br^–^, are used as the active ions for memristive switching. These cations and anions of HP tend to move in opposite directions under external electrical bias, causing self-p- and n-doping near material interfaces and resulting in enhanced carrier injection on the two sides.^29,30,47^ Additionally, the migration of HP anions may also result in halogen vacancies, which form conductive filaments inside the switching layer.^38,114,115^ Impurity ions, such as Ag and Cu, are also used as active ions within HP memristive devices.^116−120^ They undergo redox reactions and directional migration under external electrical bias to form conduction filaments, and then go through a relaxation process that reduces the conduction filaments due to the minimization of surface energy or Rayleigh instability when there is no external stimulus. These rich dynamic properties of HPs enable the realization of artificial neurons,^38,115^ artificial nociceptors,^121^ reservoir computing,^30^ synaptic plasticity,^29^ selectors,^118,122^ high-complexity computing,^47^ and even reconfigurability.^116^

### 3.3 Polymers

Polymer materials naturally consist of skeleton structures with rich vacancy sites for ion migration and embedding, as shown in Figure 5c, which can act in various types of roles, such as electroactive and inert units, electron acceptors and donors, spacer moieties of different steric effects, and nanostructures, leading to their tunable behaviors and wide applications.^71^ Additionally, the deformable and flexible properties of polymers facilitate natural and comfortable contact with biological tissue, making polymer suitable for applications in wearable devices, implantable devices, and smart surfaces.^123−127^

Polymer-based memristive devices have attracted considerable attention in recent years. Polymers such as poly(3,4-ethylenedioxythiophene):polystyrenesulfonate (PEDOT:PSS) are good proton conductors and are used to build memristive devices based on proton migration. The PEDOT:PSS film is stacked with another PEDOT:PSS film that is partially reduced with poly(ethylenimine) (PEI), i.e. PEI/PEDOT:PSS. When protons are injected from PEDOT:PSS to PEI/PEDOT:PSS, it will cause the protonation of the PEI and the removal of the holes from the PEDOT backbone, resulting in the electronic conductivity decrease of the PEI/PEDOT:PSS layer.^126^ Similarly, PEDOT:PSS is also used as a proton reservoir when stacked with WO_3_. Under an external electric field, the protons are released from PSS and injected into WO_3_, resulting in the resistive switching of WO_3_ and PEDOT:PSS.^125^ Poly(ureaurethane) (PUU) has impurity mobile cations and anions from the cross-linking reagents employed during material preparation. Under an electric field, these ions move in opposite directions and generate a temporal current response within the device.^128^ Ag is used as a major active ion to form a filament inside polymer materials, such as polymer frameworks poly(9-vinylcarbazole) (PVK),^129^ poly(vinyl alcohol) (PVA),^130^ poly(vinyl cinnamate) (PVCi),^131^ polyethylenimine (PEI),^127^ poly(ethylene oxide) (PEO),^132^ and Nafion.^133^

Though polymer-based memristive devices have undergone substantial advancements in recent years, persistent challenges revolve around the attainment of prolonged stability and reproducibility. The continuous pursuit of novel polymer materials, coupled with the refinement of device architecture and processing techniques, holds the promise of catapulting this field into further innovation and progress.

### 3.4 Biomaterials

Biomaterials, which are derived from living organisms, such as peptides, proteins, saccharides, and DNAs, exhibit unique properties and advantages over inorganic and synthetic organic alternatives. These advantages include biorealistic emulation, biovoltage operation, biocompatibility, biodegradability, sustainability, and environment-friendliness. These distinctive features make biomaterials highly compelling candidates for the development of next-generation memristive technologies.^72,73^

Biorealistic physical and chemical features are inherent in biomaterials, making them highly suitable for building components that emulate biological behaviors, such as the ion-conducting membrane as shown in Figure 5d. Researchers have used Alamethicin peptides as ion channels inserted into an artificial phospholipid membrane formed by 1,2-diphytanoyl-*sn*-glycero-3-phosphocholine (DPhPC). The doping level of the peptides within the phospholipid membrane is modulated by the electrical bias applied across the two sides of the membrane, exhibiting hysteretic doping behaviors under dynamic voltage change. The doped peptides control the migration dynamics of electrolyte ions across the two sides of the phospholipid membrane. Here, the voltage-driven peptide ion channel insertion emulates the voltage-modulated biological ion channel activation. Based on these similarities, memristive devices with synaptic behaviors and nonlinear dynamics, operating in the biological voltage range (∼100 mV) and response time (∼10 ms), have been demonstrated.^134,135^ Monazomycin is also used as a voltage-activated ion channel within a membrane formed by naturally derived porcine brain total lipid extract (BTLE) and synthetic DPhPC lipids. This shows a short-term facilitation-then-depression phenomenon emulating a biological sensory system, with different switching thresholds and hysteresis shapes in different membrane materials.^136^ Biomaterials naturally operate within biological voltage and current ranges, facilitating the creation of energy-efficient memristive devices and biocompatible interfaces with biological systems. Protein nanowires harvested from the bacterium Geobacter sulfurreducensare have been used to catalyze Ag migration within a SiO_2_ switching layer, demonstrating memristive behaviors with a switching voltage of 70 ± 3 mV, which is low enough to directly process biological voltage signals. Biovoltage artificial synapses, artificial neurons, sensory interfaces, and timing selectors have been demonstrated based on this type of switching mechanism.^137−139^

In addition to enabling biorealistic behaviors and operation range, biomaterials exhibit other unique features that can be used to build memristive devices with novel properties. Cellulose, regenerated via an environment-friendly route from cotton, allows the growth of Ag filaments and is used to build volatile memristors with biocompatibility, biodegradability, and flexibility.^140^ Tyr–Tyr–Ala–Cys–la-Tyr–Tyr (Y7C) peptide has good proton conductivity influenced by humidity, and the protons can promote Ag filament formation inside Y7C. Using Y7C as the switching material and Ag as active ions, the memristive devices show bimodal switching behavior under both electric field and humidity.^33^ DNA duplex doped with Cu ions is used to form biocompatible nonvolatile switching devices.^141^ Ultrasharp and highly ordered Ag nanocone electrode arrays have been patterned on silk fibroin obtained from cocoons, due to its thermal response under thermal scanning probe lithography, forming a highly uniform volatile memristor based on Ag migration within silk fibroin.^142^ Silk sericin has metal ion absorption properties and enables the chelation effect of Ag ions to form a conductive filament.^143^

Although biomaterials offer numerous advantages over artificial materials, they often have strict requirements for their operating environment, including the operation temperature, contact chemicals, and electric field intensity. These requirements make biomaterial-based devices difficult to incorporate with traditional CMOS circuits and prone to degrade in harsh environments.^73^ Thus, further explorations are needed to overcome these issues and expand the application field for biomaterial-based memristive devices.

### 3.5 Electrolytes

Electrolytes are materials that contain dissociated positively and negatively charged ions. They are typically in the forms of ionic liquid (IL), ion gel, or even solid-state. These materials are usually good ion conductors, allowing the charged ions to move freely inside (Figure 5e). When an electrical bias is applied across the electrolyte material, these charged ions move toward the negative and positive terminals (the cathode and anode), where they are oxidized or reduced. Extensive research and development efforts have been directed toward harnessing their properties for various energy-related applications, including fuel cells, batteries, and photovoltaic systems.

For building memristive devices, electrolytes are commonly used as a gate material to form electrolyte-gated transistors (EGTs),^74,75,144−146^ which have similar configurations to metal-oxide-semiconductor field-effect transistors (MOSFETs). The modulation of the channel conductivity is realized by the electrolyte ion accumulation on the interface of the electrolyte and dielectric layers. Due to the rich dynamics of ion migration and channel modulation, EGTs exhibit various types of memristive behaviors.

In general, three major types of EGTs have been used in memristive electronics, depending on the permeability of the semiconductor channels to electrolyte ions. The impermeable type is referred to as the electrolyte-gated field-effect transistor (EG-FET), where the channel current is regulated by the gate voltage through an ultrathin electrical double layer formed at the interface of the electrolyte gate and the semiconductor channel. Diethylmethyl(2-methoxyethyl)ammonium bis(trifluoromethylsulfonyl)imide (DEME TFSI) has been used as the electrolyte gate in contact with the MoS_2_ channel, enabling the dynamic switching of MoS_2_ conductance via the ion migration-relaxation kinetics within the ionic liquid.^147^ An ion gel layer based on poly(vinylidene fluoride-*co*-hexafluoropropylene) (P(VDF-HFP)) and the ionic liquid 1-ethyl-3-methylimidazolium bis(trifluoromethylsulfonyl)imide ([EMI][TFSI]) was used as the gate material with semiconducting indium–tungsten oxide (IWO) channels, where the ion migration-relaxation in the ion gel modulate the electronic conductance of IWO, mimicking the Ca^+^ influx in a dendritic spine.^148^ Another category is known as electrochemical transistors (ECTs), which rely on electrochemical processes of ion-permeable channel materials to tune the conductivity of transistor channels. Aqueous electrolytes with ions such as Na^+^ and Cl^–^ are used as gate materials for PEDOT:PSS channels. The injection of the electrolyte ions into the PEDOT:PSS layer changes the hole concentration via ionic-electronic charge compensation, resulting in large channel conductance modulation with S-shaped negative differential resistance (NDR) behavior.^45^ The aqueous electrolyte gate also tunes the conductivity of the ladder-type poly(benzimidazobenzophenanthroline) (BBL) via electrochemical doping by forming charged species with reduced carrier mobility, leading to a Gaussian-shaped NDR effect in the EGT transfer curve. The EGT’s dynamic behavior is also tunable by varying ion concentrations and ion types or by adding neurotransmitters and amino acids.^40^ The third type of EGTs realizes channel conductance modulation based on the mixed mechanisms of electrical double layer and electrochemical reactions. Using Li-ion conductors, such as Li_*x*_SiO_2_ and LiPON, as gate materials and amorphous niobium oxide as channel materials, the conductance modulation of the niobium oxide channels is realized via the combined mechanism of electric double layer formed at the channel interface via Li ion migration and the defect energy level generated inside the amorphous niobium oxide via Li-ion intercalation and redox reaction.^27,28^ Besides EGTs, the electrolyte can also be used directly as switching materials between electrodes. For example, a water-based electrolyte containing 3,4-ethylenedioxythiophene (EDOT) and benzoquinone (BQ) as oxidizing and reducing species shows dendritic electropolymerization behavior under AC electrical input to form PEDOT:PSS conductive fibers, emulating the structural plasticity of biological neuron network.^149^

Compared with other ionic memristive devices, the electrolyte-based memristive devices have ultralow voltage operation, resulting in high energy efficiency of neuromorphic processing.^27,150−152^ In addition, their combined ionic-electronic conductivity makes them feasible to mimic complex neuromorphic plasticity.^27,28^ Moreover, the structural versatility of EGTs enables flexible configurations to receive multiple inputs^27,147−149,153^ or generate multiple outputs.^154^

### 3.6 Synthetic Nanostructures

Besides different types of thin film materials, researchers also aim to use synthesized nanostructures to facilitate ion migration paths within the switching layers (Figure 5f), which may offer better performance or unique properties. These nanostructures, ranging from quantum dots, nanowires, nanosheets, nanorods, nanotubes, etc., provide ion migration paths due to the defects and boundaries near them.

Nanorods, nanosheets, nanoparticles, and quantum dots have surfaces or spacers that interact with the switching ions and facilitate ion migration. SiO_2_ nanorods have pockets that allow Ag electromigration along the nanorod surface.^76,77^ Oxidized MXene nanosheets have TiO_2_ particles serving as spacers to provide paths for Ag migration.^155^ Au nanoparticles are self-assembled and anchored with anions, while the N(CH_3_)_4_^+^ cations move through the gaps of the nanoparticles, modulating the conductance by tuning the local equilibrium of counterions.^78^ Nitrogen-doped graphene oxide quantum dots act as an ionic conductor with numerous functional groups interacting with Ag cations during migration.^156,157^ InP-ZnS quantum dots also allow Ag to migrate under an electric field.^158^

Single nanowires, nanotubes, nanobelts, and nanoshells can also be used to build memristive devices. These structures usually exhibit semiconductor properties, and under voltage stimulation, the interface of the nanostructure and metallic electrode is doped, or the nanostructure itself is doped with ions, to facilitate electron conduction. Single ZnO nanowires can induce memristive switching via trap occupation and oxygen vacancy migration.^159^ Single TiO_*x*_ nanobelts also show gradual volatile switching behavior, believed to be achieved by oxygen vacancy migration resulting in trap redistribution that tunes the Schottky barrier and leads to the trap-assisted quantum tunneling.^48^ Single nonstoichiometric CuO nanowires show threshold-switching behavior which is believed to be due to Cu filament formation and rupture.^160^ Single MoS_2_/MoO_2_ structures have a conductive MoO_2_ inner core and an insulative MoS_2_ shell matrix, allowing Ag ions to migrate through the insulative shell layer.^54^

Networks of conductive nanowires that are coated with insulating layers are used to build planar memristive devices with dynamic switching behavior via ion migration through the intersection of the nanowires. Ag nanowires are used by coating with polymers or polycrystalline materials that have migration paths, such as PVC,^161^ PVP,^162,163^ P(VDF-HFP),^164^ and TiO_2_.^165,166^ Nanowires of silver compounds, such as Ag_2_Se^167^ and AgI,^168^ also show hysteresis switching behavior via the formation of metallic conductive filaments under voltage stimulation. The Ag nanowire network can also realize switching behavior via the Ag nanoparticle migration between the separated nanowire fragments.^169^

The migration paths provided by the nanostructures facilitate ion migration for high-speed and low-energy devices. The nanostructures can be highly ordered during synthesis or via self-organization, providing ordered paths for the ion to migrate, reducing the randomness during filament growth, and improving the uniformity of the switching process. Moreover, the network topology of nanowires leads to complex dynamic switching for high-order dynamic computing or high-dimensional encoding. Single nanowires or nanotubes enable controllable migration path building at the nanoscale, leading to reproducible nanoscale memristive devices.

### 3.7 van der Waals Materials

Compared to traditional memristive devices based on bulk materials, the research on memristive devices based on van der Waals (vdW) materials is still in the early stages. Vacancies in vdW materials can provide active host sites for cations or anions to form conductive filaments. Grain boundaries in vdW crystals can also serve as fast pathways for cation and anion migration, enabling low-activation-energy switching without an electroforming process (Figure 5g). The interlayer spaces and surfaces of vdW materials may serve as ion migration pathways in some cases (Figure 5h). The structural defects and compositional imperfections in van der Waals materials are highly tunable by magnetic field, electric field,^170^ heat treatment,^171^ etc., providing various approaches to modify the switching performance of memristors. Furthermore, high-quality vdW materials provide atom-level uniformity and are promising to effectively reduce the device-to-device and cycle-to-cycle variation,^79^ which is a common issue of memristive devices based on bulk materials.

Most vdW memristive devices are based on metal ion migration within their grain boundaries or vacancies. Hexagonal boron nitride (hBN) allows the migration of metal ions via its defects and grain boundaries. Ag and Cu are reported to show threshold-switching behavior within hBN by penetrating the hBN layer via these local defects and forming conduction filaments inside.^172−174^ Due to the excellent insulation of hBN, atomically thin hBN with single-atom defects is used to build threshold switching devices with high scalability, where the switching is realized by the formation and relaxation of Ag atomic point contact at the defect location.^175^ Atomically thin graphene is stacked with atomically thin hBN to form a heterostructure switching layer, where Ag filaments are confined by the graphene defects, enhancing the threshold switching performance such as ON/OFF ratio and leakage current.^176^ Sulfur vacancies in CuInP_2_S_6_ (CIPS) also allow Ag cations to migrate and form conduction filaments, demonstrating both short-term and long-term switching behaviors.^177^ Ti_2_C_3_ (MXene), in the form of two-dimensional flakes, can build a switching layer with ion migration paths between the flakes. Cu ions are demonstrated to migrate and form conduction filaments within MXene for threshold-switching.^178^

The surface and interlayer spaces of vdW materials are also utilized for metal ion migration. A type of diffusive memristors based on a lateral Ag/hBN/Ag structure switches by the diffusion of Ag^+^ ions along the surface of the hBN layer and the formation of volatile conduction filaments due to the low energy barrier of Ag diffusion.^179^ Volatile resistive switching behaviors in lateral Ag/MoS_2_/Ag devices are attributed to the formation of conduction filaments due to the migration of Ag cations along the MoS_2_ channel surface with the help of sulfur vacancies in MoS_2_.^180^ Memristive switching behavior in a MoS_2_ layer can also be induced by Li-ion intercalation. The Li ions move between MoS_2_ layers under electric fields, and the concentration change of Li ions leads to the transition of MoS_2_ between 2H (semiconductor) and 1T’ (metal) phases.^181^

The migration and redistribution of defects and vacancies also result in resistive switching of vdW materials. Grain boundaries and vacancies of MoS_2_ are demonstrated to migrate under an external electrical bias, modifying the Schottky barrier height at the metal–semiconductor interface, resulting in diverse resistive switching behaviors^170,182^ The motion of oxygen vacancies during SET and RESET operations in oxidized MoS_2_ is observed under HRTEM and utilized to build oxygen-vacancy-based 2D memristors with high-temperature stability.^183^ Oxygen vacancy migration within insulative graphdiyne oxides (GDYO) leads to the formation of a conductive graphdiyne (GDY) filament, while the oxygen-deficient region is unstable and the GDY filament is volatile.^184^

## 4 Applications of Ion-Based Memristive Devices

IMDs have been employed to emulate (instead of simulate) a wide range of biological behaviors due to their rich ion dynamics. Among these, the emulation of neuronal and synaptic behaviors is particularly crucial, as they are fundamental components of all types of neuromorphic computing algorithms. Moreover, recent studies have demonstrated that incorporating dendritic and reservoir computing elements can enhance computational efficiency and versatility. Additionally, IMDs have shown promise in applications as selectors and in hardware security, which are also vital for the advancement of emerging computing systems. Each of these applications is related to different dynamic properties discussed in Section 2. Neuronal behaviors usually include leaky-integration processes, based on ion assembly and relaxation with comparable speeds, and firing events, based on the large nonlinearity inside the IMDs. Synaptic behaviors involve long-term nonvolatile memory, due to slow ion relaxation in a long-time range, and short-term dynamic processes, due to fast ion relaxation in a short-time range. Dendritic behaviors are mainly about spatiotemporal leaky integration of the input signals, which results from ion assembly and relaxation with comparable speed. Reservoir behaviors require the nonlinear integration of the input signal, which results from the cooperation of assembly, relaxation, and nonlinearity of the IMDs. Selector applications focus on the fast switching and large ON/OFF ratio of the devices, which are based on fast assembly and relaxation speed and large nonlinearity. Hardware security applications generally utilize the stochasticity of IMDs. The realization of these applications using IMDs is thoroughly discussed in this section.

### 4.1 Neuronal Behaviors

**Figure 6:**
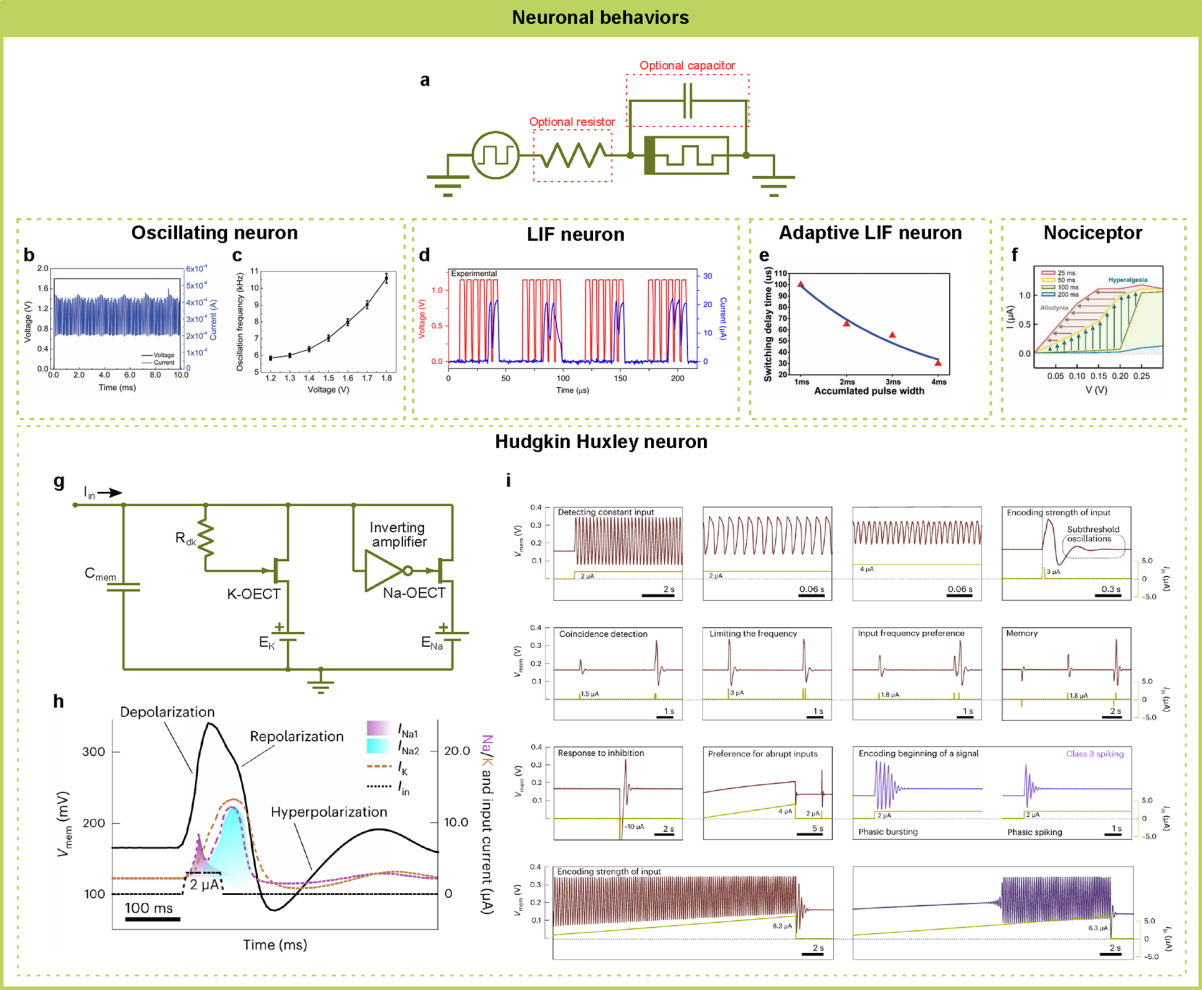
Neuronal behaviors realized by IMDs. (a) A single IMD, sometimes with its parasitic capacitor, a parallel capacitor, or a series resistor, can be used to realize many types of neurons, including (b, c) oscillating neurons, (d) leaky-integrate-and-fire neurons, (e) Adaptive leaky-integrate-and-fire neurons, and (f) nociceptors. (g) By using several IMDs with complex auxiliary electrical components, a Hodgkin-Huxley neuron can be realized, showing the biorealistic (h) action potential and (i) spiking behaviors. (b, c) Reproduced with permission from ref (190). Copyright 2022, Wiley-VCH. (d) Reproduced with permission from ref (64). Copyright 2018, Springer Nature. (e) Reproduced with permission from ref (66). Copyright 2023, Wiley-VCH. (f) Reproduced with permission from ref (121). Copyright 2024, Wiley-VCH. (g–i) Reproduced with permission from ref (40). Copyright 2023, Wiley-VCH.

IMDs are promising candidates for replicating both the fundamental processes and functionalities of biological neurons (Figure 6). In these devices, the dynamics of electrolyte ions across neuron membranes in biological cells can be emulated by the dynamics of active ions within the switching materials. The different ion concentrations in extracellular and intracellular fluids in biological neurons can be effectively mimicked by electrodes with varying chemical potentials. The functionalities of memristive devices enable artificial neurons to emulate the generation and transmission of electrophysiological signals observed in biological neuronal systems.^185,186^ This capability paves the way for developing neuro-inspired computing systems tailored for future AI applications.^187^

Neuronal oscillations are repetitive or rhythmic neuronal activities commonly observed in biological nervous systems, which are crucial for brain functions like signal encoding, feature binding, information transmission, memory formation, computation, etc.^188,189^ A single Te/TiTe_2_/Pt memristive device can show neuronal oscillation behaviors under constant voltage biases with the oscillation frequency tuned by the voltage amplitude. This is enabled by the special electrochemical-thermal properties of Te ions, which assemble and form conduction filaments via electrochemical reactions, while the filaments melt easily under joule heating due to the low thermal conductivity and low melting point of Te, phenomenologically emulating the functions of biological Na^+^ and K^+^ ion channels,^190^ as shown in Figure 6b and 6c. More commonly, an oscillatory neuron is formed by an ion-based memristor in series with a load resistor.^191,192^ There exists an RC circuit composed of the memristor parasitic capacitance and the load resistor, which is used to integrate the input electronic signals and control the intervals between output firings. The ion-based memristor acts as a threshold-switching device to fire output spikes when the integrated charge is large enough on the parasitic capacitance.

Leaky-integrate-and-fire (LIF) neurons are the most commonly used elements in the field of neuromorphic computing. This behavior captures the essential aspects of a biological neuron: leaky integration of the input charge and then firing spikes at a certain threshold voltage while maintaining sufficient simplicity and efficiency. Consequently, there has been significant interest in constructing LIF neurons using novel devices. IMDs are intrinsically suitable for building LIF neurons, with ion assembly enabling charge signal integration, ion relaxation allowing signal leakage, and nonlinear switching behaviors realizing fast threshold firing. Various ion-based memristors are used as LIF neurons following such a concept, based on ion migrations of Ag^64,129,143,193,194^ or Cu^92,178^ (Figure 6d). In some cases, the ion-based memristors are used for threshold switching and current leakage, while the charge integration is performed by a parallel capacitor,^103^ for current inputs, or by an input resistor and a parallel capacitor,^39,137,195,196^ for voltage inputs. A resistor may also be added in series with the memristor to convert output current spikes into voltage spikes.^76,115,158,197−199^ Another resistor may be added in parallel with the capacitor to control the leakage speed.^200,201^ Ion-based memristors are also combined with CMOS components to realize LIF neurons with more functionalities, such as refractory period and cascaded circuit connection.^202−204^

In addition to LIF behavior, intrinsic plasticity, i.e., short-term memory, is another important neuronal feature observed in biological neurons and useful in SNN algorithms. Ag ions form aggregates with slower relaxation dynamics compared with the fast relaxation of thin Ag ion filaments, leading to a short-term threshold-lowering effect even after the memristor is fully switched off. Based on these phenomena, LIF neurons with intrinsic plasticity have been demonstrated using diffusive memristors composed of Au/yttria-stabilized zirconia with Ag doping (Ag:YSZ)/Au,^66^ with the adaptive firing threshold as shown in Figure 6e, and a stacked phase change memristor and diffusive memristor structure composed of TiW/Ge_2_Sb_2_Te_5_ (GST)/Ag:SiO_2_/Ag/Au.^205^

Ion-based memristors are also used to build nociceptors, a type of sensory neuron that responds to noxious stimuli and provides warnings to the central nervous system. The major properties of nociceptors are allodynia, the decrease of detection threshold; and hyperalgesia, the increase of response intensity, under abnormal stimulations, as shown in Figure 6f. These properties align well with the nature of ion migration within switching materials. Under large external stimulations, the accumulation of active ions within IMDs results in a higher background concentration, which decreases the device switching threshold, strengthens conduction channel, and increases the output intensity. Therefore, artificial nociceptors based on various active ions and switching materials have been demonstrated, including Ag ion in SiO_*x*_,^80^ ZrO_*x*_,^89^ TiO_*x*_,^81^ MAPbI_3_,^121^ hBN,^179^ SiO_2_ nanorod,^77^ and silk fibroin;^142^ Br ion in MAPbBr_3_;^38^ and oxygen vacancies in Nb_2_O_5-x_/Al_2_O_3-y_ heterostructure.^206^ The artificial nociceptors are connected with thermal sensing resistors and pressure sensing resistors under a constant voltage source to demonstrate artificial thermoreceptors^80,206^ and artificial mechanoreceptors.^121^ By connecting with a triboelectric nanogenerator, a self-powered Mechano-Nociceptor has also been demonstrated.^179^

The Hodgkin–Huxley (H–H) neuron model^207^ is renowned for its more accurate representation of the dynamic processes in biological neurons compared to LIF. Thus, an artificial H–H neuron could be very useful for building systems that emulate the realistic behavior of biological neuron networks. Previous researchers have used the NDR effect of Mott memristors with resistors, capacitors, and voltage sources to build an artificial H–H neuron.^208^ NDR effects are also observed in IMDs, especially EGTs. Using two EGTs to emulate the biological ion channels, a parallel capacitor and two voltage sources to emulate the biological membrane capacitance and ion concentration differences, the H–H neuron circuit can demonstrate various neural features, including tonic spiking, latency, integration, refractoriness, resonance, threshold variability, rebound spike, accommodation, phasic bursting, phasic spiking, spike encoding, stochastic spiking, and chemical-modulated spiking,^40^ as shown in Figure 6g to 6i. Using two EGTs with some passive resistors and capacitors, the constructed neuron emulated some nonlinear dynamics and bifurcations similar to the H–H neuron.^45^

To achieve the desired neuron behavior, many aspects of requirements need to be considered for the IMDs, among which the major ones are the firing energy, the operation speed, the plasticity, the dimension, and the stochasticity, as shown in Figure 12a. The firing energy is usually represented by the energy required to fire an output spike. The operation speed is represented by the fastest input speed that can be handled by the neuron. The plasticity refers to the time range of the short-term memory effect of the device. The dimension is determined by the feature size of the device. The stochasticity is related to the randomness of the device behavior, which is usually characterized by the variation of threshold voltage. These aspects not only determine the capability of IMDs to achieve high-performance artificial neurons but also measure the potential of IMDs to emulate biological neurons. The dynamics of active ions in existing IMDs already cover most of the range of biological neurons (Figure 12a).^56,209−212^ However, there are still no artificial neurons based on IMDs that can simultaneously achieve all the requirements. For example, no IMDs are satisfying the low energy consumption and long intrinsic plasticity of biological neurons (Figure 12d). This is possibly due to the fact that most of the previous efforts have been focused on the development of LIF neurons, while artificial neurons with intrinsic plasticity are still not well explored. More slow dynamics similar to the effect of high Ag ion background concentration in YSZ may be observed in IMDs in the future. Limited by the current and voltage detection resolution of available measurement instruments, the dynamics of IMDs at extremely low voltage (<10 mV) and current (<1 nA) have not been sufficiently explored. By amplifying these signals via peripheral circuits, it would be possible to utilize low voltage and current dynamics to build highly energy-efficient neurons.

### 4.2 Synaptic Behaviors

The fundamental synaptic characteristics of biological neural systems entail the diffusion dynamics inherent in biological processes. Biological synapses rely on the modulation of calcium ion (Ca^2+^) dynamics across a biological membrane and neurotransmitter dynamics within the synaptic cleft. When an action potential reaches a presynaptic neuron, it triggers the influx of Ca^2+^ ions, which initiates the release of neurotransmitters. The presynaptic and postsynaptic stimuli tune the synapse’s sensitivity to the Ca^2+^ ions and result in the changes of excitatory postsynaptic potential or current (EPSP or EPSC) under the same stimulus, leading to the memory effect of the synapses, which serves as the foundation for both learning and information storage within biological neural systems. Thus, to build biorealistic computing systems, it is essential to replicate the adaptive behaviors of synaptic plasticity with neuromorphic devices.

**Figure 7:**
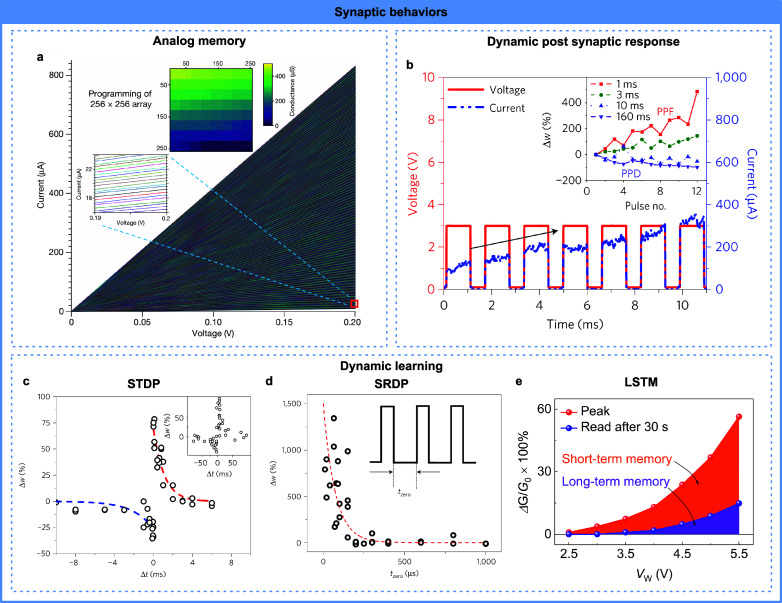
Synaptic behaviors realized by IMDs. A single IMD can show (a) analog memory,^213^ (b) dynamic postsynaptic responses,^41^ and dynamic learning rules such as (c) STDP,^41^ (d) SRDP,^41^ and (e) LSTM.^27^ (a) Reproduced with permission from ref (213). Copyright 2023, Springer Nature. (b–d) Reproduced with permission from ref (41). Copyright 2017, Spring Nature. (e) Reproduced with permission from ref (27). Copyright 2023, Springer Nature.

Analog long-term memory is the most central property of synapses. The conductance of IMDs is determined by the conduction channels formed by active ion migration, which are intrinsically analog processes. Thus, nonvolatile ion-based memristors are highly suitable for building analog long-term memory synapses. Ag and Cu ions are utilized to demonstrate the coexistence of analog memory and short-term memory effects by tuning the switching conditions.^92,94,129,173^ Proton migration within proton-conducting phosphosilicate glass (PSG) realizes analog memory with nanosecond programming speed.^214^ Oxygen vacancies exhibit long-term stability within Ta/Al_2_O_3_:TiO_*x*_/HfO_2_/Pt devices and are able to achieve state-of-the-art 2048 conductance levels after denoising,^213^ as shown in Figure 7a.

Paired-pulse facilitation (PPF) and paired-pulse depression (PPD), which are considered forms of short-term plasticity, have been widely observed during synaptic transmission.^215,216^ Specifically, when a sequence of successive stimuli reach a presynaptic neuron with brief intervals, the synaptic response to the latter stimulus is either amplified or diminished relative to the former depending on the interval length.^217^ These phenomena are believed to contribute to information processing and network synchronization in the nervous system.^218^ With the assembly and relaxation dynamics of active ions, nearly all types of IMDs show PPF and PPD behaviors,^29,41,92,102,128,134,137,159,169,219−221^ with Figure 7b as an example, especially under mild stimulation conditions that result in weak conduction channels.

The interaction of the long-term and short-term effects results in dynamic learning capabilities within the IMDs. Spike-timing-dependent plasticity (STDP) represents a form of long-term synaptic plasticity that governs the modification of synaptic weights in response to the timing relationship between pre- and postneuron spiking activities.^222^ According to this principle, the strength of synaptic connectivity can be adjusted according to the temporal correlation between pre- and postneuron firing patterns. Joule heating, in combination with oxygen vacancy diffusion within tantalum oxide, has been utilized to demonstrate a second-order memristor capable of exhibiting STDP behavior.^51^ The long-term plasticity of a halide perovskite memristor, based on its native ion migration, exhibits a nonlinear correlation with the stimulation frequency, enabling STDP behavior within a suitable input time range.^47^ Ag ion migration inside TiO_2_ is shown to form partial filaments after the first pulse, allowing either SET or RESET processes depending on the following pulse polarity, resulting in STDP behavior.^81^ Volatile ion-based memristors have also been used in combination with nonvolatile memristors to demonstrate the STDP behavior^41,131,134,205^ as shown in Figure 7c. In addition, some researchers have engineered the pulse shapes of applied voltages, rather than exploiting device dynamics, to achieve STDP behaviors, which will not be discussed here. Besides STDP, spike-rate-dependent plasticity (SRDP)^41,51,78,81,131,134^ (Figure 7d) and the transition from short-term memory to long-term memory under repeated stimulations^28,54,98,117,130,155,221,223−226^ (Figure 7e) have been demonstrated with IMDs, both of which originate from the gradual assembly of ions under external stimuli.

The key factors in evaluating a synaptic device are shown in Figure 12b, involving switching energy, long-term memory, dimension, and dynamic range. The switching energy is the energy required to switch the device on and off. The long-term memory is the retention time of the device conductance states. The dimension is represented by the feature size of the device. The dynamic range refers to the analog conductance range the device can operate in. These factors are important for achieving biorealistic computing. Besides, the short-term memory of IMDs, represented by the time length of the devices showing fast conductance change, also plays an important role in emulating the temporal behaviors observed in biological synapses. The artificial synapses composed of IMDs exhibit a wide variety of behaviors and encompass most of the biological synapse properties (Figure 12b).^212,216,227−229^ However, the trade-off between retention time and switching energy of memristive devices makes it challenging to realize IMDs that are comparable to biological synapses in terms of both long-term memory duration and device switching energy (Figure 12e). While nonvolatile memories with long endurance exist, they do not demonstrate the dynamic behaviors necessary to enable the transient properties and learning algorithms of biological synapses. To achieve low switching energy, further IMD exploration at lower current, voltage, and conductance ranges is necessary.

### 4.3 Dendritic Behaviors

In addition to the emulation of somatic and synaptic behaviors, the dendritic dynamics of neurons have been proposed to be crucial for achieving highly efficient computing in biological brains.^230^ This extends from the soma and connects to multiple neurons via synapses. It not only integrates the signals received from these synapses but also ranks and preprocesses these signals through its intrinsic dynamic processes before sending the signals to the soma, thereby enhancing the computing capability of the biological neuron.^231^ Accordingly, neuromorphic learning algorithms based on dendritic behaviors have been developed to improve neural network performance on spatiotemporal data sets.^232^ Developing devices that can realize these dendritic behaviors efficiently is therefore important.

**Figure 8:**
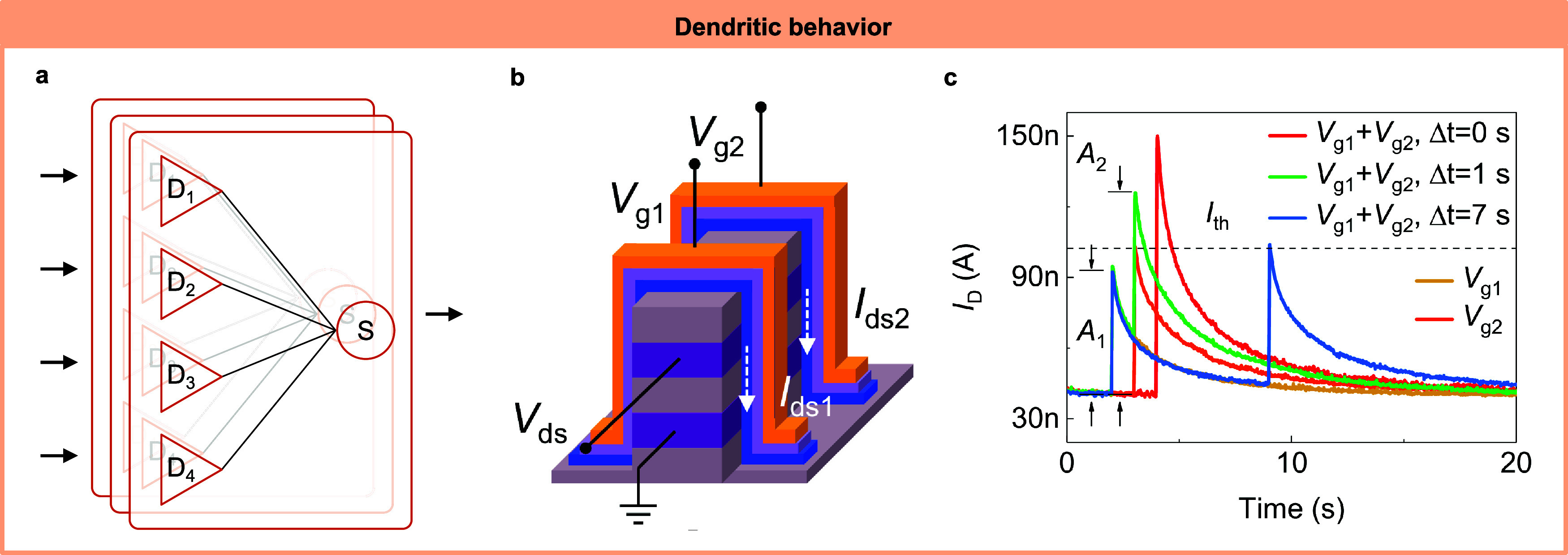
Dendritic behaviors realized by IMDs. (a) The schematic of the dendritic device application. Various dendritic devices receive and process inputs and send outputs to the soma for further processing.^101^ (b) An ion-based memristive transistor can be used as a dendritic device.^27^ It can integrate spatial information using its multiple gate terminals and process temporal information based on its ion dynamics, demonstrating (c) the spatiotemporal information processing capability. (b, c) Reproduced with permission from ref (27). Copyright 2023, Springer Nature.

From the temporal perspective, dendrites integrate input signals with a decay, which can be emulated by the assembly and relaxation dynamics of ion-based memristors. In addition, the input signals from multiple synapses interact within a dendrite dynamically, known as spatiotemporal signal processing or heterosynaptic behaviors, which can be emulated by controlling the ion dynamics within IMDs via the interaction of input voltage biases from multiple electrodes (Figure 8a). Thus, IMDs are promising candidates for realizing artificial dendrites.

The Ag-based diffusive memristor can realize temporal signal integration modulated by ion assembly dynamics. By stacking the diffusive memristor on top of a transistor, one can form a neuro-transistor that can be connected to multiple synapses to realize dendritic spatiotemporal signal processing.^202^ Similarly, the transport of oxygen ions in oxide materials can achieve dynamic assembly that provides filtering functions of dendrites. Multiple oxygen-based dynamic memristors are connected to a Mott memristor, which is used as a threshold firing soma that sums the signals from the dynamic memristors, to build an artificial dendrite.^69,101^ The movement of Li^+^ between MoS_2_ 2D layers can be manipulated by the signals from multiple electrodes simultaneously, which naturally realize the heterosynaptic behavior of dendrites.^181^ For light-sensitive materials, such as GeSe_2_ or N-GOQDs, the movement of Ag ions can be controlled by both electrical signals and optical stimulations, so the voltage input and light stimulation can be treated as excitatory and inhibitory inputs of a heterosynaptic structure to generate the postsynaptic current, which emulates the behaviors of biological dendrites.^156,233^ Memristive transistors are intrinsically suitable for introducing multiple gate electrodes on one device for multiple inputs; and with the temporal memory effects under ion migration, the devices can demonstrate the spatiotemporal processing capabilities of dendrites straightforward,^27,234^ as shown in Figure 8b and 8c.

Dendrite devices are not commonly required in traditional neural networks but are playing an important role in realizing biorealistic computing. To realize a promising dendrite device, the operation voltage and the operation current need to be minimized for high efficiency. Besides, input density, device dimension, and the time resolution of a dendrite device are also very important for real applications. The input density is calculated from the number of inputs one device can handle in a unit area. The dimension is represented by the feature size of the device. The time resolution is determined by the input speed that the device can handle. These factors are summarized in Figure 12c for various dendrite devices. The development of artificial dendrites is still in its early stage and the current dendritic devices are still far from matching the performance of biological dendrites (Figure 12c).^235−238^ Besides low energy efficiency due to the large operation voltage and current range, which is a similar issue faced by artificial synapses and neurons, the input density and the operation speed of the artificial dendrite are also important factors that need to be simultaneously optimized (Figure 12f).

### 4.4 Reservoir Computing

**Figure 9:**
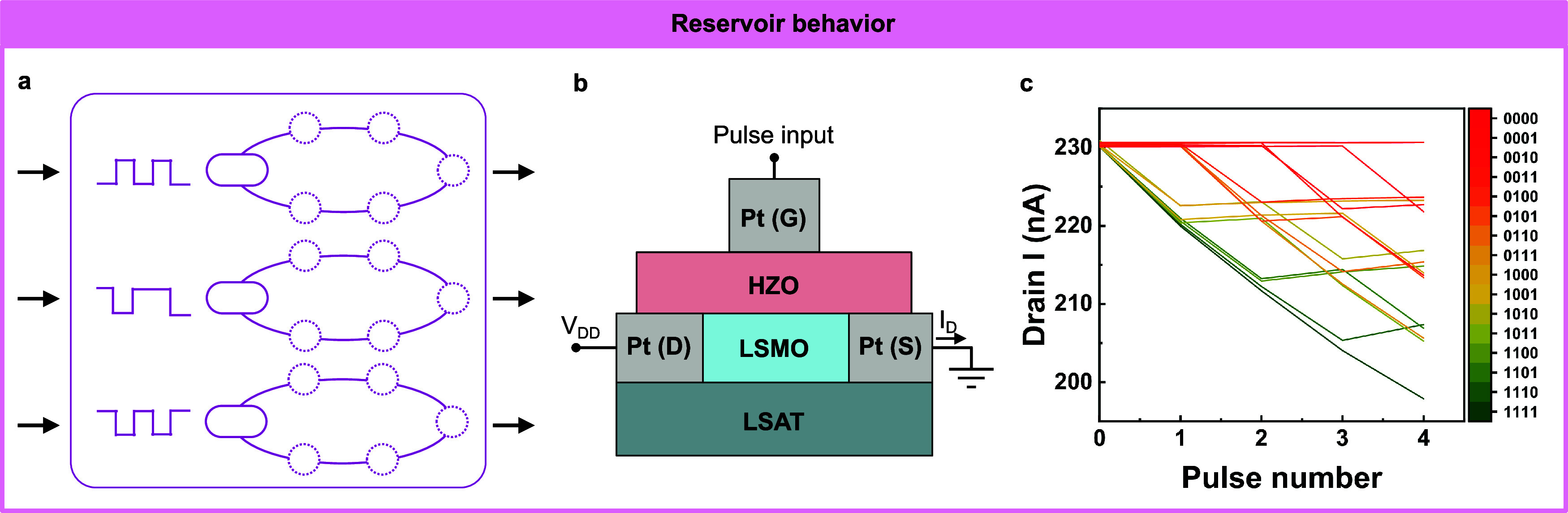
Reservoir behaviors realized by IMDs. (a) Schematic of using IMDs as reservoirs.^55^ Mask signals are usually used before the IMDs to capture different information from the input signals. Then the IMDs evolve their status under the stimulations to generate output signals. (b) Migration of oxygen vacancies from HZO to LSMO can be used to demonstrate reservoir behaviors.^245^ (c) With the evolution of channel conductance under different pulse sequences, the final output drain currents are different. (a) Reproduced with permission from ref (55). Copyright 2022, Springer Nature. (c) Reproduced with permission from ref (245). Copyright 2023, Springer Nature.

Reservoir computing (RC) is a highly efficient network for processing temporal signals due to its low training cost compared to standard recurrent neural networks.^239^ A physical device suitable for the hardware implementation of RC systems must possess two key properties: short-term memory and nonlinear dynamics, enabling the nonlinear change of device status under electrical stimuli to transform the input signals into a high-dimensional feature space.^161,240−244^ IMDs, with their nonlinear dynamic ion migration processes, are ideal for reservoir implementation. Moreover, a single IMD is capable of emulating the function of a reservoir network, enabling the incorporation of high-density reservoirs into a neuromorphic computing system (Figure 9a). This makes IMD one of the most promising candidates for reservoir implementation. To be used as a reservoir, it is desired that the IMD show more number of states within its operation range.

WO_*x*_ memristors based on oxygen vacancy drift and diffusion effects were used as reservoir devices. RC systems of WO_*x*_ memristor arrays were experimentally used for image and handwritten digit classification tasks, achieving high recognition accuracies.^241,246^ Oxygen vacancy migrations between Hf_0.5_Zr_0.5_O_2_ (HZO) and La_0.67_Sr_0.33_MnO_3_ (LSMO) are used to tune the memristive transistor status, resulting in reservoir computing systems for various spatiotemporal classification tasks^245^ (Figure 9b and 9c). Diffusive memristor devices based on Ag ion migrations were used for reservoir computing with fast time step (τ < 5 ms), demonstrating MNIST handwritten digit classification tasks.^240^ Furthermore, by combining the Ti/TiO_*x*_/Pd device with peripheral circuits, the reservoir dynamics can be arbitrarily tuned and accurately reproduced, achieving a fully hardware reservoir analog computing system with real-time processing capabilities.^55^ Besides using a single device as a reservoir to process temporal data, self-organized resistive switching networks of nanowires have also been developed as more intricate reservoir systems to achieve both spatial signal and temporal signal transformation inside the reservoir.^161^ Reservoir networks based on polymer-coated silver nanowires were constructed for image classification and time-series prediction tasks. The reservoir states are represented by the topological conductivity map of the junctions formed by intersected nanowires, which intrinsically possess high complexity and nonlinearity that benefit the reservoir performance.

### 4.5 Selectors

**Figure 10:**
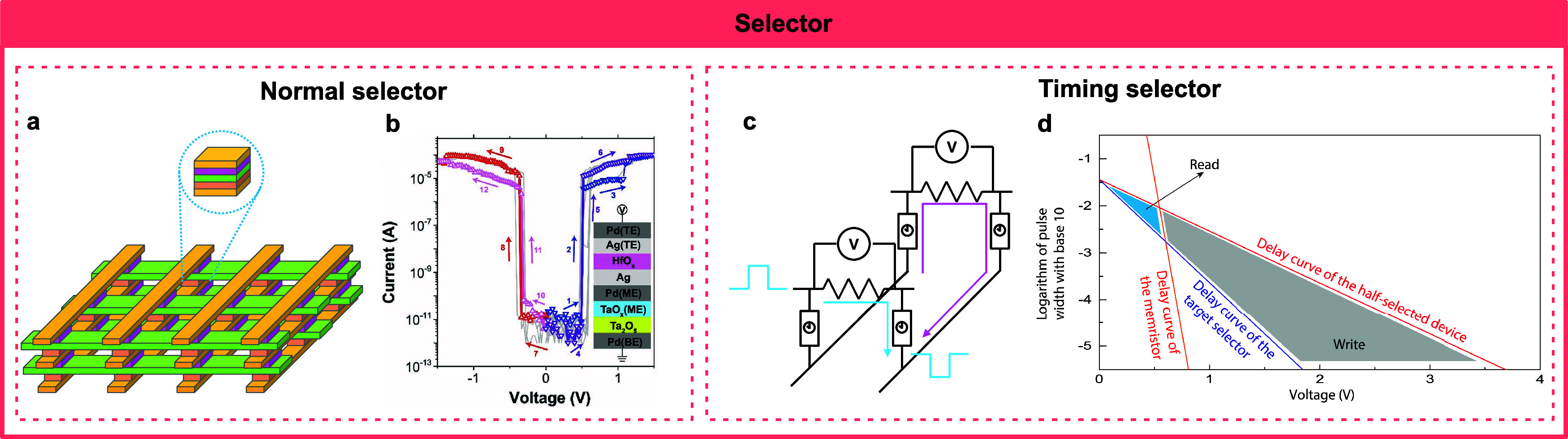
Selectors realized by IMDs. (a) The IMD can be used as a selector with threshold-switching behavior to decrease the sneak path current in a memristor crossbar array. (b) The IMD is connected in series with a nonvolatile memristor to form a 1S1R structure, where the current below the IMD threshold is limited.^42^ (c) The transient behavior of the IMDs can be utilized to build a selector with timing features.^252^ (d) Both the pulse amplitude and the pulse width determine the read and write range of the selector. (b) Reproduced with permission from ref (42). Copyright 2017, Wiley-VCH. (c, d) Reproduced with permission from ref (252). Copyright 2022, Wiley-VCH.

To operate memory devices in the form of a crossbar array, a major challenge is the sneak path current during resistance programming and reading processes. When applying a voltage between the target row and column to change the resistance state of a memristor, the current also unintentionally flows through other lines, which influences the accuracy of the readout and disrupts the unselected or partially selected devices. This compromises the operational reliability and energy efficiency of the memristive crossbar arrays. There are several ways to tackle this problem, such as the one transistor-one memristor (1T1R),^247−249^ one diode-one memristor (1D1R),^250,251^ and one selector-one memristor (1S1R) configurations, among which 1S1R is promising to achieve the most compact structure by vertically stacking the selectors and memristors (Figure 10a). Besides, single devices like self-selective and self-rectifying memristors are also attractive due to their simpler structures.

The selector needs to possess high nonlinearity and large current density for effective programming operations and reduced energy consumption. The ON/OFF ratio, also known as the dynamic range, of the device is an important factor that needs to be considered. Besides, the operation speed, the reproducibility, and the device feature size are other factors that are generally evaluated for selector devices. IMDs, with their large ON/OFF ratio and fast volatile switching dynamics enabled by the nonlinear dynamic ion migration, have shown great promise in selector applications. The formation and rupture of Ag filaments inside ceramic switching materials are the most used methods to achieve IMDs for selector applications, due to the high mobility, fast redox reaction rate, and diffusion dynamics of the Ag ions, as well as the good insulation and localized migration paths within the ceramic materials. Among these devices, Ag migration within ALD-grown HfO_2_ has achieved the highest ON/OFF ratio (>10^10^) with a response time of <250 ns,^42,253^ which are used to form 1S1R blocks (Figure 10b). In addition, Ag has also demonstrated an ON/OFF ratio of 10^4^ to 10^9^ in other ceramic materials, including SiO_2_,^254^ ZnO,^255,256^ and MgO.^83^ Moreover, the rich dynamics of Ag migration inside amorphous SiO_2_ have also resulted in the demonstration of timing selectors^252^ (Figure 10c and 10d). A timing selector is a novel concept that functions based on the highly nonlinear relation between switching time (or assembly dynamics) and applied voltage, in contrast to traditional selectors based on their nonlinear current and voltage relationships. Other than thin film ceramics, nanostructures,^76^ halide perovskites,^115^ and polymers^131^ have also been used to build Ag selectors, although with inferior performance for selector applications, achieving satisfactory for other applications. Cu is also used as the active ion in HfO_2_ for selectors. Although the performance is not on par with that of the Ag devices due to its relatively low mobility, the Cu device is compatible with the CMOS processes.^91^ Other efforts have also been made to improve the IMD selector performance, such as using an AgCu alloy as the active electrode to improve the thermal stability,^200^ using Te as the active electrode to enhance the current density,^223^ etc. More improvement schemes will be discussed in Section 5.

### 4.6 Hardware Security

**Figure 11:**
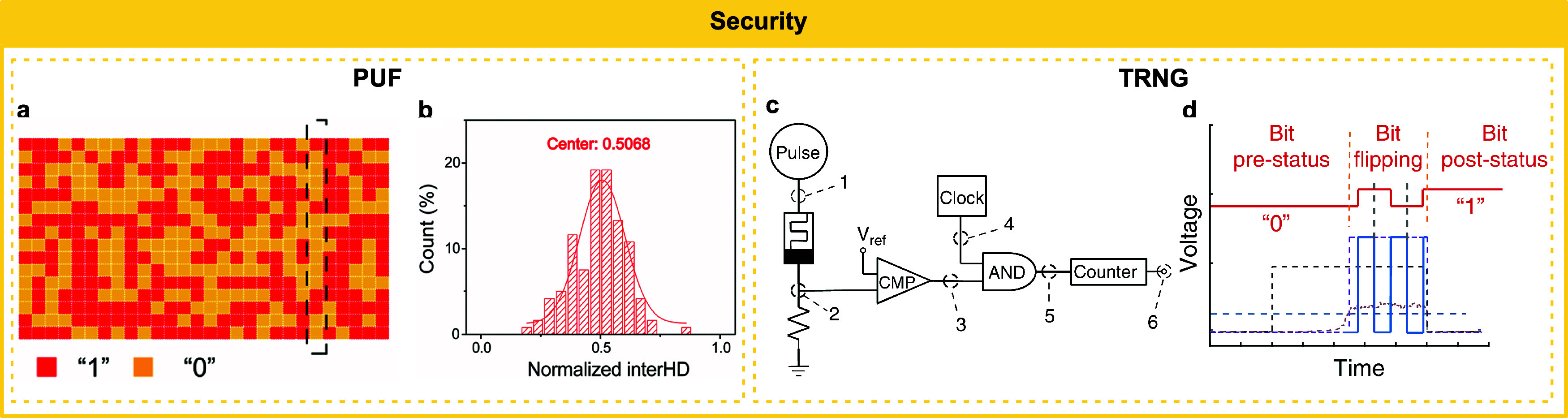
Security applications based on IMDs. (a) IMDs with random switching threshold form 28 4-by-4 IMD arrays. Under the same switching voltage, the switched devices are represented in red, and the nonswitched devices in yellow. (b) To show the uniqueness of the PUF, the interclass Hamming distances between each two 16-bit strings generated by the 4-by-4 arrays are summarized and fitted to Gaussian distribution. (c) The random switching behaviors of the IMDs can also be used to form a circuit for TRNG. (d) Whether the output bit flips or not is determined by whether the IMD switches on or not, leading to the final random output. (a, b) Reproduced with permission from ref (257). Copyright 2018, Royal Society of Chemistry. (c, d) Reproduced with permission from ref (67). Copyright 2017, Springer Nature.

Physical unclonable function (PUF) devices are promising hardware security primitives for device authentication and key generation, characterized by their intrinsically unclonable physical operations. They are used to define a unique mapping from “challenges” to “responses” that serve as the unique identifier for IoT devices. The reproducible and controllable stochasticity of the devices is the most desired feature for hardware security applications. Besides, energy efficiency and scalability are two factors that are also essential for hardware security to achieve large-scale and integrated implementations. IMDs show intrinsic device-to-device variations due to various factors, such as ion distribution, defect morphology, and thickness nonuniformity, which are usually undesired for computing applications. However, these physical variations can be utilized to realize PUF naturally with IMDs. By tuning device fabrication recipes, the ratio of the switchable Au/Ag:SiO_2_/Au devices at certain voltage conditions can be adjusted. A large array of these devices forms a PUF that can generate 16-bit binary maps with desired randomness,^257^ as shown in Figure 11a and 11b. In addition, a PUF based on W/Ag/MgO/Ag/W has been demonstrated to show similar randomness of switchable and nonswitchable devices. Furthermore, this PUF is flexible and transient, capable of dissolving in water, making it suitable for more security requirements.^258^

True random number generators are becoming increasingly important with the development of IoT as they can be used to generate random number sequences for hardware security. Since the ion dynamics within IMDs are intrinsically stochastic (Figure 3), these devices are naturally suitable as a good source for random numbers (Figure 11c and 11d). The randomness of ion dynamics can be characterized by different traits during device operation, and each aspect may be utilized to generate random numbers. The assembly time of an Ag/Ag:SiO_2_/Pt device is stochastic and tunable with different voltages. Under the same voltage pulse stimulus, the ON-time of a device with a large current is used to determine the transition times of an output value between ‘0’ and ‘1′, as shown in Figure 11c and 11d. The final output sequence passes the standard randomness test (NIST 800-22 test suite).^67^ The same method has been used to demonstrate a high-speed TRNG using a Cu_0.1_Te_0.9_/HfO_2_/Pt device based on Cu migration.^259^ The switching probability for whether the device can be switched on or not under a short voltage pulse is also a good source of randomness. By inverting the circuit output based on the switching times of the memristive device Ag/TiN/HfO_*x*_/HfOy/HfOx/Pt under a sequence of short pulses, the final output results also pass the randomness test.^260^

**Figure 12:**
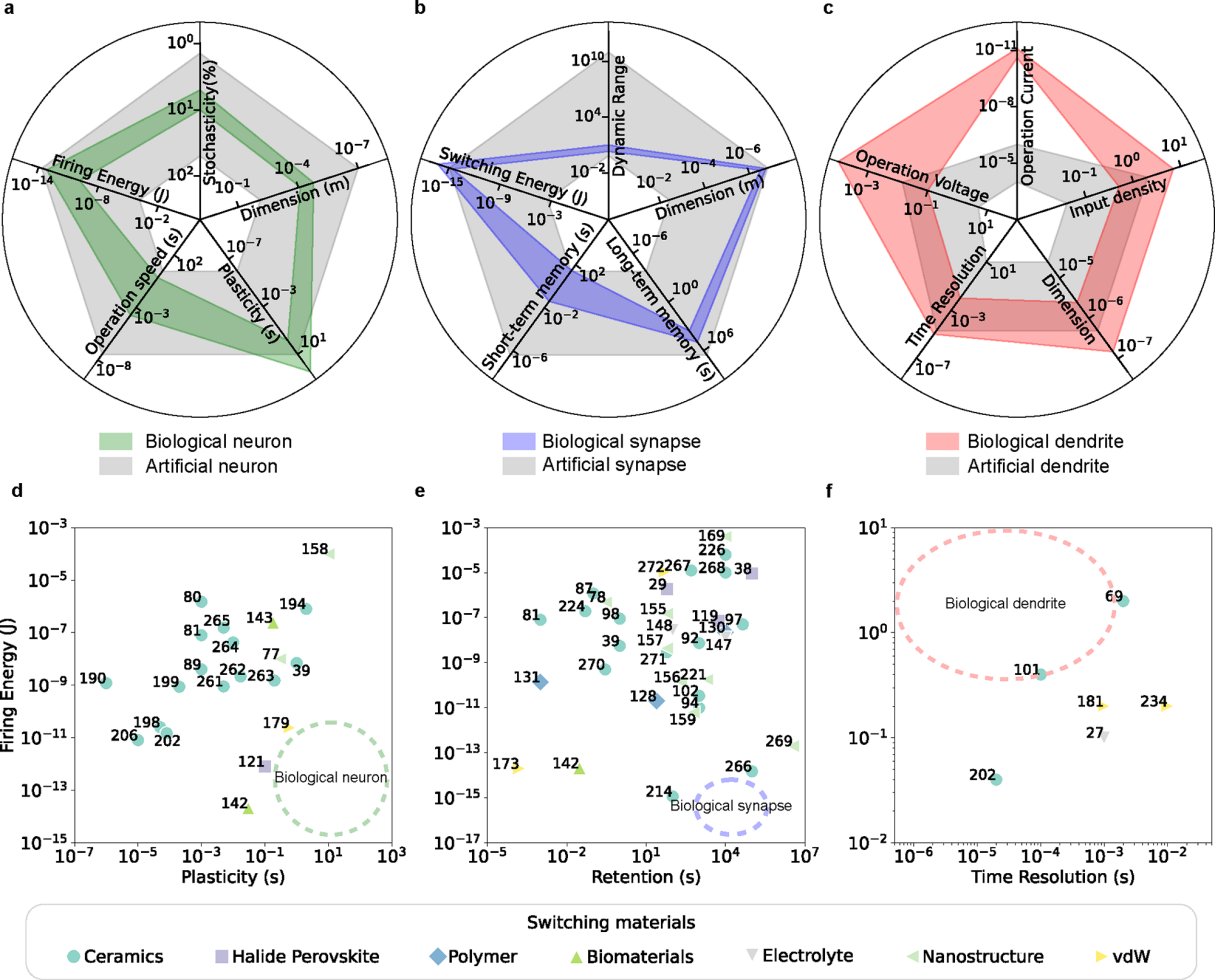
(a–c) The performance of the artificial neurons, synapses, and dendrites is summarized and compared with their biological counterparts. The maximum and minimum values achieved by the IMDs are indicated by the gray areas, while the maximum and minimum values observed in the biological systems are indicated by the colorful areas. It is worth noting that the best performance parameters of the artificial devices are collections of different devices rather than being obtained in a single best device. (d–f) Two representative properties of the artificial neurons (refs (39, 77, 80, 81, 89, 121, 142, 143, 158, 179, 190, 194, 198, 199, 202, 206, and 261−265)), synapses (refs (29, 38, 39, 78, 81, 87, 92, 94, 97, 98, 102, 119, 128, 130, 131, 142, 147, 148, 155−157, 159, 169, 173, 214, 221, 224, 226, and 266−272)), and dendrites (refs (27, 69, 101, 181, 202, and 234)) are selected for further comparison. The values observed in the specific IMDs, shown as the colorful markers, are compared with the values observed in the biological systems, shown as the colorful dashed circles.

## 5 Guide to Designing Ion-Based Memristive Devices

The comparisons between artificial synapses, dendrites, and neurons to their biological counterparts show that the IMDs are promising to emulate BNN behaviors and realize biorealistic computing, but there are still gaps between the artificial and biological components in several specific properties that need to be simultaneously enhanced. To provide a guide, we review and summarize what may be considered when developing IMDs. They are divided into the aspects of active ions, switching layers, electrodes, band engineering, and device geometry. The details of each aspect are discussed in the following sections.

### 5.1 Active Ion Selection

**Figure 13:**
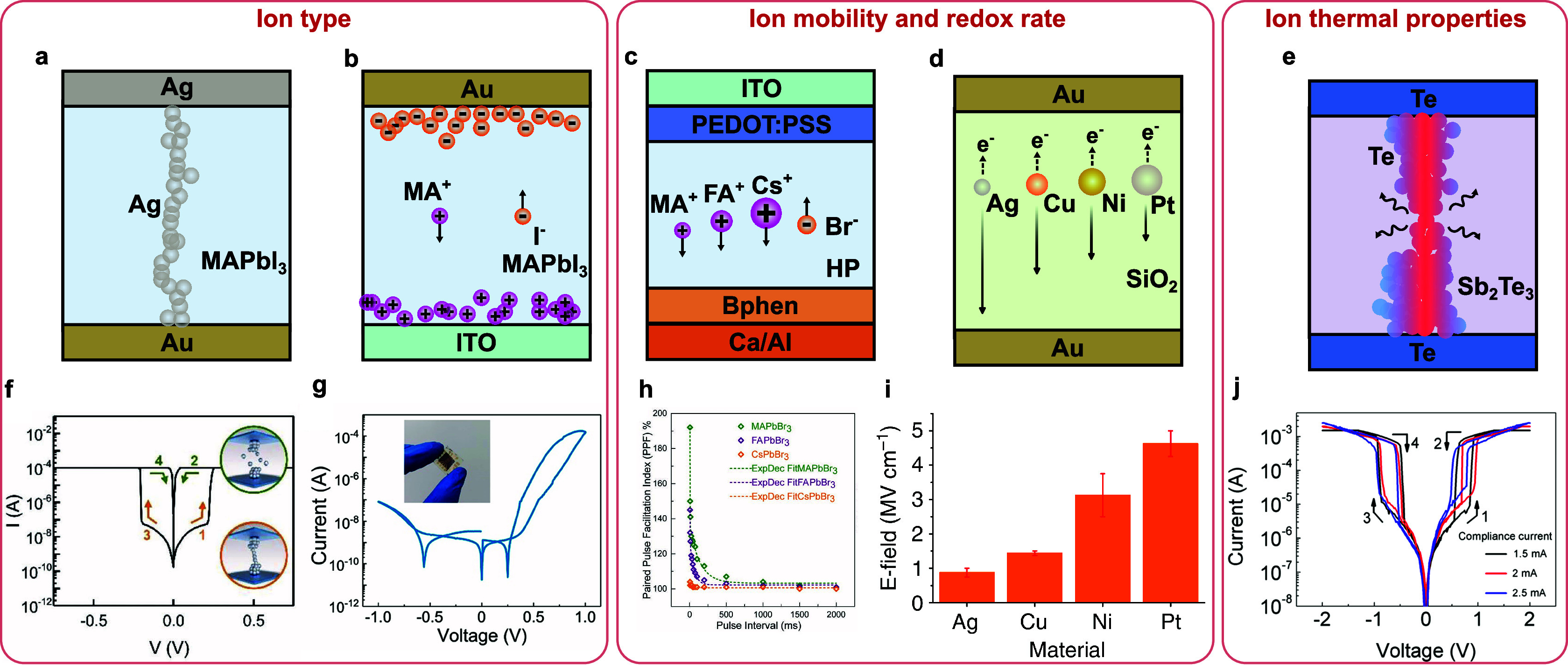
Ion modifications of IMDs. (a, f) Metal ions tend to aggregate and result in filamentary switching with sharp switching slope.^121^ (b, g) Native ions tend to distribute evenly and result in nonfilamentary switching with gradual slope.^30^ (c, h) Ions with different mobilities result in different switching behaviors.^29^ (d, i) Ions with different mobilities and redox reaction rates result in different thresholds of switching electric fields.^26^ (e, j) The unique thermal properties (low thermal conductivity and low melting point) of the Te ions lead to the easy melting of Te filaments under large currents.^223^ (f) Reproduced with permission from ref (121). Copyright 2024, Wiley-VCH. (g) Reproduced with permission from ref (30). Copyright 2022, American Chemical Society. (h) Reproduced with permission from ref (29). Copyright 2018, Wiley-VCH. (i) Reproduced with permission from ref (26). Copyright 2014, Springer Nature. (j) Reproduced with permission from ref (223). Copyright 2021, Springer Nature.

The switching mechanism of an IMD is highly related to the type of its active ions (Figure 13). Different ion types show different interfacial energies and lead to distinct switching dynamics. The memristors with electrochemically active ions, such as Ag, Cu, Te, etc., tend to form filaments during switching because these metal atoms congregate together (Figure 13a). The filamentary structure is confirmed by in situ electron microscopy during the switching processes of the metal-ion-based memristors.^35,41^ This results in significant conductivity changes and very large ON/OFF ratios.^41,273^ The filament tip also enhances the electric field intensity, speeding up the ion migration and resulting in the fast SET switching of the device, as shown in Figure 13f.^42,121^ The off-switching speed of filamentary memristors is fast as well, since the rupture of a thin filament is a highly localized process.^42^ However, due to the randomness of filament formation and the dependency of filament properties on local environments, the devices usually show large C2C and D2D variations and need other types of enhancement to tackle the randomness issues. On the other hand, if the interfacial energy between the active atoms is close to or even larger than that between the active atom and the switching medium, the switching atoms tend to distribute homogeneously in the medium (Figure 13b). This usually results in nonfilamentary switching.^274^ For metal oxide-based memristors, if the migration of oxygen vacancies is localized and forms a conductive filament, they are called filamentary-type memristors. In contrast, if the oxygen vacancies accumulate at the interface, they are called interfacial-type memristors, which are nonfilamentary.^275,276^ Oxygen vacancies in TiO_*x*_ are confirmed to show nonfilamentary switching with an area-dependent ON-conductance. They show slower switching dynamics compared to filamentary memristive devices and are more suitable for temporal information processing applications.^39^ The migration of native ions in Halide Perovskites also generally leads to nonfilamentary conduction channels and gradual switching behaviors,^30,277^ as shown in Figure 13g. Due to the mild and uniform switching processes, the D2D and C2C variations of the devices are small. Hydrogen cations lead to nonfilamentary switching as well, enabling reconfigurability^43^ and uniformity^214^ with gradual switching dynamics.

The selection of active ions also determines the electron transport mechanism in the ON state of the device, as already explained in Figure 2. Ohmic conduction is observed in most metal IMDs and is usually desired for computational applications that require linear current–voltage relations. If the conducting clusters (e.g., regions doped by ions) do not fully bridge the switching layer, the device conduction may involve tunneling, exhibiting nonlinear current–voltage characteristics. The interface-dominant conduction mechanism usually shows self-rectifying current–voltage characteristics that are typically attributed to a Schottky-like interfacial barrier that is beneficial for mitigating the sneak path currents when used in a crossbar array.^100^ The conduction mechanisms based on carrier migration across traps formed by ion defects involve complex hopping and tunneling behaviors and may demonstrate high-order dynamics.^206,278^ Device switching based on the modulation of transistor channel conductance via electric double layers minimizes the gate leakage current and favors high energy efficiency.^27,28^ The modulation of transistor channel conductance via electrochemical reactions with the ambient ions also enables the applications of chemical sensing and neuromorphic interface.^40,45^

Ion mobility is another important factor influencing the dynamics of both the assembly and relaxation processes of IMDs. Different ions have vastly different mobilities, determined by migration barrier or activation energy during ion migration,^279^ making it possible to fine-tune the dynamics of the memristive devices by changing active ions (Figure 13c). For example, Ag usually shows a higher mobility than Cu due to its lower activation energy, and this leads to the lower switching energy and faster switching speed of Ag compared to Cu.^200^ The migration barriers of MA, FA, and Cs cations are different in halide perovskites, which are used to realize the short-term memory dynamics with slightly different response speeds,^29^ as shown in Figure 13h. For those devices involving redox reaction during filament growth, the redox reaction rate is another important factor that influences the filament growth mode and filament morphology in cooperation with the ion mobility^26^ (Figure 13d and 13i), and controls the switching properties. For example, low ion mobility and high redox-reaction rate make the filament thick near the active electrode and thin near the inert electrode, facilitating the filament rupture near the inert electrode, leading to fast relaxation,^42^ and the filament residue near the active electrode, leading to second-order dynamics.^66^ With low ion mobility and low redox reaction rate, Ru tends to form nanoclusters within the switching layer, which leads to low conductance at the ON status for low-energy analog computing.^266^

The thermal response of the active ions also plays an important role in memristive switching. Te filaments yield volatile switching under a large current compliance but nonvolatile switching under a small current compliance, which is the reverse of the normal cases, providing a solution to achieve a long state lifetime with a small energy input^190,223^ (Figure 13j). The poor thermal conductivity and low melting point of Te make the filament dissolve under large currents (Figure 13e).

Among the various active ions used in IMDs, silver has been the most widely utilized due to its compatibility with a broad range of switching materials and its versatility across numerous applications, as summarized in Table 1. This preference arises from several advantageous properties of Ag. First, it can be introduced into most matrix materials and remains stable within them. Additionally, Ag exhibits high activity and diffusivity, making it easy to induce switching behaviors, as manifested by the lowest volatile switching thresholds achieved with Ag-based IMDs.^137^ This high activity also enables Ag IMDs to exhibit diverse dynamic behaviors to realize various applications. Furthermore, Ag filaments tend to spontaneously rupture at zero bias driven by the minimization of the interfacial energy between Ag and dielectric materials, resulting in reliable volatile switching. Nevertheless, filamentary switching typically exhibits more variability and stochasticity than nonfilamentary switching mechanisms. In addition, most mobile ions, including Ag, are not CMOS-friendly. CMOS-compatible ions, such as protons, are preferred when CMOS integration is needed.

**Table 1:** Summary of the IMDs Based on Different Types of Active Ions

Ion types	Applicable Switching Materials	Applicable Applications	Smallest Volatile Switching Threshold (V)	Largest Volatile Switching Endurance (Cycle)	CMOS Compatibility
Silver	Ceramic,^42^ vdW,^173^ Biomaterial,^139^ Halide Perovskite,^116^ Nanostructure,^76^ Polymer^142^	Synapse,^129^ Neuron,^64^ Selector,^42^ Dendrite,^264^ Reservoir,^280^ High-order complexity,^66^ Near-sensor,^121^ In-materia,^161^ Reconfigurable,^116^ Hardware Security^67^	0.06^137^	1 × 10^8^ ^42^	Not compatible^281^
Copper	Ceramic,^282^ vdW^178^	Synapse,^94^ Neuron,^178^ Selector,^91^ Reservoir,^95^ Near-sensor,^259^ Hardware Security^259^	0.26^90^	1.8 × 10^6^ ^263^	Compatible with diffusion barrier^283^
Lithium	vdW,^181^ Electrolyte^28^	Synapse,^181^ Neuron,^28^ Dendrite,^181^ Near-sensor,^28^ Reconfigurable^28^	∼1.5^28^	4 × 10^4^ ^181^	Compatible with diffusion barrier^284^
Tellurium	Ceramic^99^	Synapse,^223^ Neuron,^190^ Selector^223^	1.15^190^	5 × 10^4^ ^223^	Compatible^285^
Oxygen	Ceramic,^55^ vdW^184^	Synapse,^39^ Neuron,^103^ Selector,^184^ Dendrite,^69^ Reservoir,^55^ Near-sensor^51^	0.50^286^	1 × 10^6^ ^39^	Compatible^287^
Halide Anion	Halide Perovskite^38^	Synapse,^38^ Neuron,^38^ Selector,^115^ Near-sensor^115^	0.52^115^	1 × 10^6^ ^115^	Compatible with protection layer^288^
Proton	Perovskite Nickelate,^43^ Polymer^125^	Synapse,^214^ Near-sensor,^43^ Reconfigurable^43^	NA	1.6 × 10^6^ ^43^	Compatible^289^

### 5.2 Switching Layer Modification

**Figure 14:**
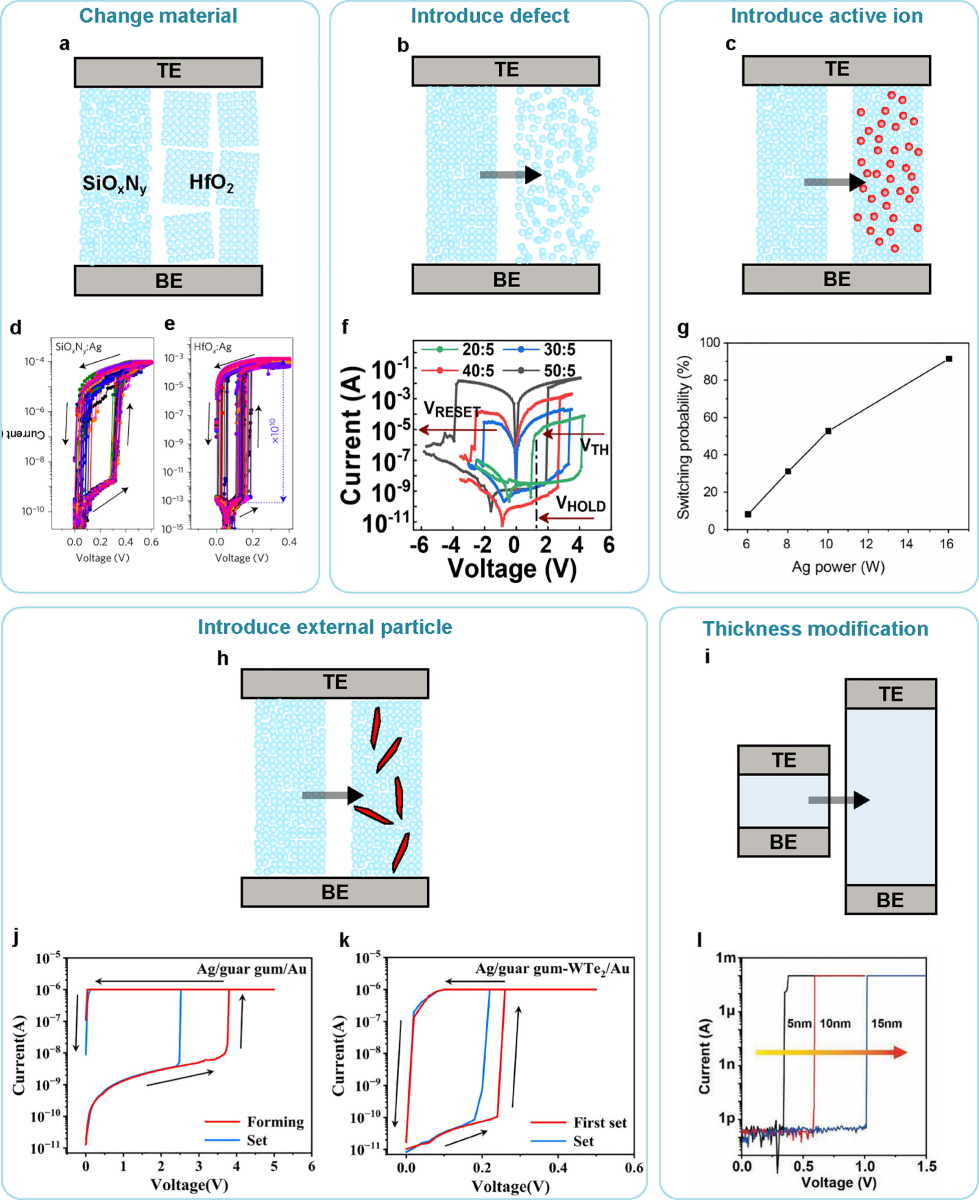
Switching layer modifications of IMDs. (a, d, e) The change of switching materials leads to changes in switching behaviors.^41^ (b, f) The argon-to-oxygen ratio during switching layer deposition is changed to tune the defect densities within the switching layer, leading to changes in the switching behaviors.^194^ (c, g) Active ions are co-sputtered with the switching layer, and more active ions make the device easier to switch.^257^ (h, j, k) External WTe_2_ nanosheet particles are doped into the switching layer, leading to forming-free IMDs with lower OFF conductance.^221^ (i, l) With the increasing switching layer thickness, the switching threshold also increases.^192^ (d, e) Reproduced with permission from ref (41). Copyright 2017, Springer Nature. (f) Reproduced with permission from ref (194). Copyright 2022, IEEE. (g) Reproduced with permission from ref (257). Copyright 2018, Royal Society of Chemistry. (j, k) Reproduced with permission from ref (221). Copyright 2023, IEEE. (l) Reproduced with permission from ref (192). Copyright 2019, Wiley-VCH.

As discussed in the previous sections, different types of switching materials are utilized to build IMDs with different physical, chemical, and electrical properties (Figure 5). This results in their various switching dynamics with distinct strengths and weaknesses. The morphologies of the switching materials also affect different switching properties^41^ (Figure 14a, 14d, and 14e). Ceramic materials are usually good insulators with dense structures, which facilitate low OFF conductance and large ON/OFF ratios. In addition, they can also be made very thin due to their low leakage currents, which shortens the migration path of ions and enables fast switching.^42^ However, the grain boundaries of ceramics are distributed randomly, enlarging the variations from device to device.^290^ Halide perovskite materials with their special lattice structures enable the facile migration of the active ions, which leads to gradual but rich dynamic switching behaviors^29,30,38,47^ for reconfigurable devices.^116^ Biomaterials usually possess low operation voltages and currents, making them suitable for low-energy applications.^134,137^ However, halide perovskites and biomaterials usually require special treatments during their preparation, which are not compatible with conventional CMOS fabrication. Electrolyte materials provide free spaces for ion migration, which makes it difficult to form stable conduction channels inside, but by using them as transistor gate materials, the rich ion dynamics within the electrolytes can be reflected by the channel conductance changes of the devices, enabling complex and repeatable switching dynamics.^27,28,147,148^ Switching layers based on synthesized nanostructures possess rich migration paths, which could lead to fast and efficient switching performances,^157^ uniform switching behaviors,^76−78^ or complex switching dynamics.^161,164,167^ VdW materials, with their atomic thicknesses and highly crystallized structures with local defects, are promising to achieve minimum device sizes, high switching speeds, good uniformities, and some unique properties.^173,175,176^

The defect density of the switching layer, which can usually be tuned during the fabrication of devices, is another important factor that influences the IMD switching behavior.^291^ A higher defect density leads to more migration paths and lower ion migration barriers, facilitating ion migration and filament growth, and usually resulting in lower switching voltage and faster switching speed. However, high defect densities may also result in uncontrolled filament growth, with large nonuniformity and low endurance. On the contrary, a switching layer with a low defect density makes it harder to form a filament, leading to higher switching voltage and thinner filament. The thin filament ruptures easily and thus facilitates reproducible volatile switching behavior and large device endurance. Besides, the confined and localized filament due to fewer defects improves the device uniformity in some cases. By tuning the Ar:O_2_ flow rate ratio during the growth of the switching layer TaO_*x*_, the density of oxygen vacancies is tuned, and the device changes from volatile switching to nonvolatile switching with the increasing of oxygen vacancies^194^ (Figure 14b and 14f). Some post-treatments may also change the defect density levels within the switching materials. Annealing makes the switching layer material crystallize and form grain boundaries to facilitate filament growth,^266^ increasing the device ON conductance. Ar plasma treatment increases the vacancies inside the switching layers, leading to forming-free devices with fast and uniform switching behaviors.^261^ γ Radiation increases the defect densities within the SiO_2_ by introducing silicon nano inclusions (NIs), which lowers the Ag migration barriers and results in abrupt switching with low voltage thresholds.^195^

The switching dynamics can also be tuned by doping the active ions directly into the switching layers. Predoped ions lead to forming-free devices, avoiding the random and overinjected filaments during the drastic forming processes and leading to better device uniformity and endurance. The doped ions may also decrease the threshold voltages and lead to easier switching operations. The drawback is that a high-level ion doping induces a higher OFF conductance of the device with a larger leakage current.^82^ Rapid thermal annealing (RTA) lets Ag diffuse into the HfO_2_ switching layers, resulting in forming-free devices with high uniformity.^253^ The co-sputtering technique is used to dope Ag into HfO_2_ and leads to forming-free devices with better nonlinearity and lower OFF conductance.^292^ Cu is doped into HfO_2_ via annealing to enable forming-free threshold switching.^91^ Decreasing the Oxygen gas pressure during HfO_2_ sputtering increases the density of oxygen vacancies, which are used to form filaments in the memristive devices, leading to forming-free devices.^286^ Co-sputtering of Ag and GST tunes the OFF-conductance of the device.^220^ By depositing the switching layer on top of Ag, Ag will diffuse spontaneously into the switching layer. The devices with self-doped Ag tend to show double-switching behavior (i.e., the device jumps from low conductance to middle conductance and then from middle conductance to high conductance) during the SET process.^225^ Ag is doped in the MgO switching layer via spontaneous diffusion from the Ag electrode layer. The ion doping level can also be tuned by the thickness of the silver layer and further tunes the switching probability of the memristive devices.^258^ Ag doping level in SiO_2_ is tuned by tuning the Ag deposition power during co-sputtering, which also tunes the switching probability of the devices,^257^ as shown in Figure 14c and 14g. Co-sputtering Ag with HfO_2_ forms Ag clusters in HfO_2_ after annealing, resulting in devices with better uniformity.^293^ Ion doping can also be achieved by depositing the switching layer via electrochemical deposition in mixed solutions, for example, a mixture of AgNO_3_ solution and Zn(NO_3_)_2_·6H_2_O solution, to achieve devices with a good uniformity.^85^

Adding certain materials into the switching layers is another way to tune the properties of the switching layers. The special properties of the added materials usually influence the active ion dynamics and tune the switching behaviors. WTe_2_ nanosheets are added into guar gum switching layers to act as conductive islands, which lead to forming-free devices with low threshold voltages and good uniformity,^221^ as shown in Figure 14h, 14j, and 14k. Tetracyanoquinodimethane (TCNQ) molecules are added to the polymer framework to coordinate with the Ag atoms, thus, serving as a migration path for Ag ions inside the polymer framework and leading to low threshold voltages, good uniformity, and high endurance of the devices. Besides, TCNQ molecules dissolved in solvents with different polarities show different distributions and domain sizes, resulting in different filament sizes and switching dynamics.^129^ Protein nanowires can act as a catalyst inside the SiO_2_ switching layers, which facilitates the Ag reduction for low threshold voltages and fast switching speeds.^137,139^

The switching layer thickness will influence the switching behavior as well. Thinner switching layers enhance the electric field intensities and shorten the filament growing paths, leading to low switching voltages with fast and uniform filament growth. However, as a potential drawback, thin switching layers make devices easy to be electrically shorted and may reduce the yield and endurance of the devices. HfO_2_ switching layer thickness is tuned via ALD growth time, leading to devices with various threshold voltages,^59,192^ as shown in Figure 14i and 14l. The thickness of the SiO_2_ switching layer is also tuned via ALD growth time and influences both the threshold voltage and switching uniformity.^294^ Tuning the polymer switching layer thickness between Ag nanowires via annealing the device under different temperatures results in different threshold voltages and OFF conductances.^163^

### 5.3 Switching Layer Combination

**Figure 15:**
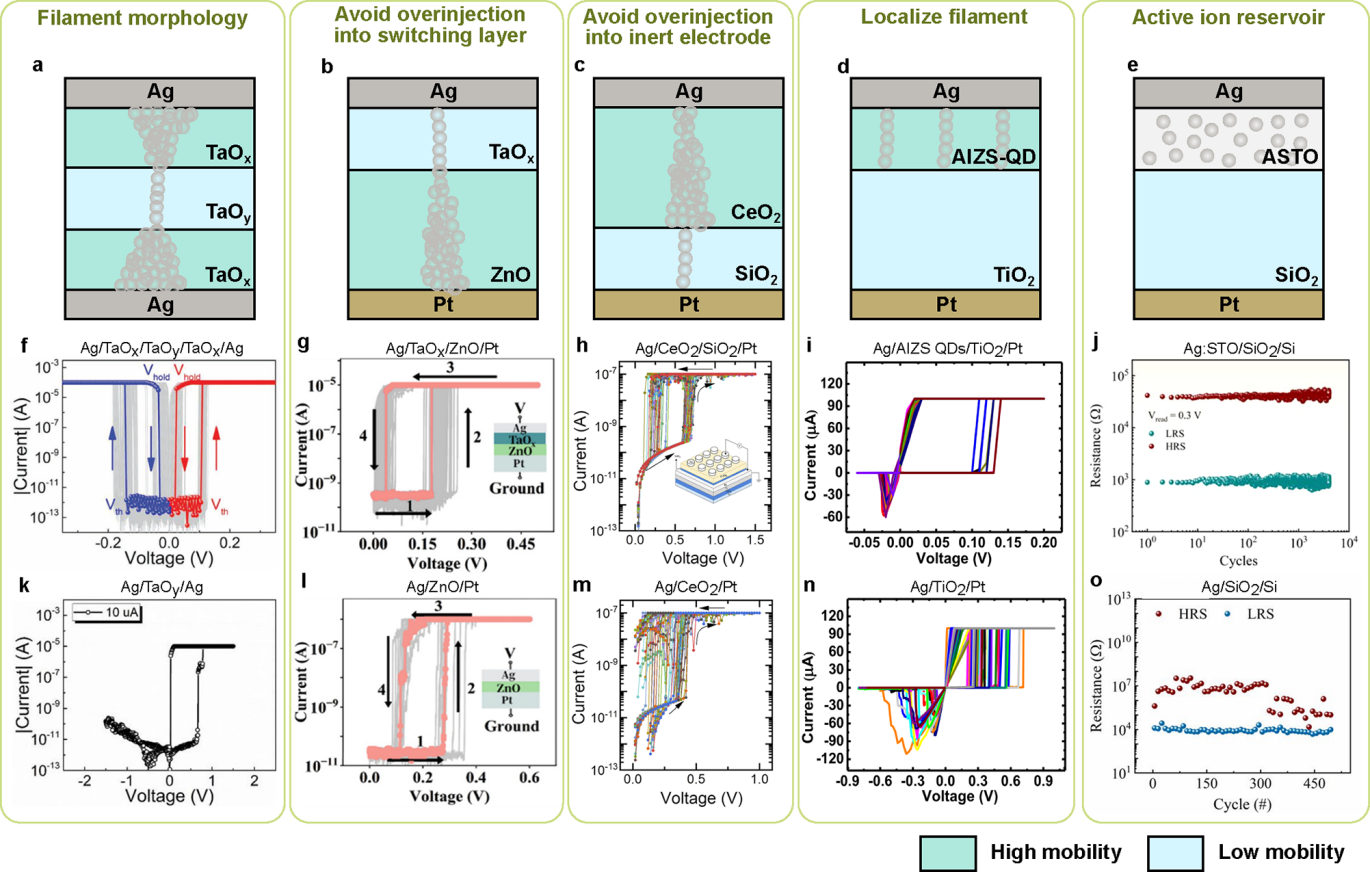
Switching layer combinations of IMDs. (a) The middle low-mobility switching layer sandwiched by two high-mobility switching layers leads to the hourglass-shaped filament, showing (f, k) lower switching voltage, larger ON/OFF ratio, and faster switching speed.^296^ (b, c) A low mobility switching layer in contact with the active electrode^300^ (the inert electrode^301^) avoids the overinjection of the active ions into the dielectric layer (the inert electrode), resulting in (g, l, h, m) better uniformity and endurance. (d) A high mobility layer in contact with the active electrode facilitates the localized filament formation inside the high mobility layer, which acts as the sharp electrode during switching and leads to (i, n) better uniformity and lower switching voltage.^268^ (e, j, o) The insertion of a switching layer doped with the active ions results in better uniformity and endurance.^226^ (f, k) Reproduced with permission from ref (296). Copyright 2019, Wiley-VCH. (g, l) Reproduced with permission from ref (300). Copyright 2024, American Institute of Physics. (h,m) Reproduced with permission from ref (301). Copyright 2022, American Institute of Physics. (i, n) Reproduced with permission from ref (268). Copyright 2021, American Chemical Society. (j, o) Reproduced with permission from ref (226). Copyright 2022, American Chemical Society.

Besides using a single material as the switching layer, researchers seek to use heterogeneous multilayer structures, with different ion mobilities and redox reaction rates in different switching mediums, to realize precise and intricate control of the ion dynamics (Figure 15).

If several types of materials are used together, due to their different ion mobilities and redox reaction rates, the filament growth behavior and morphology can be designed to fit specific needs.^295^ Different filament shapes are achieved with this method, like jar-shaped, cone-shaped, and inverted-cone-shaped, to serve different purposes. Besides, the different ion dynamics within the switching layers introduce high-order dynamics to the devices for complex neuromorphic computing applications. A three-layer structure TaO_*x*_/TaO_*y*_/TaO_*x*_, with the middle layer more oxygen-rich and insulating, leads to an hourglass-shape filament and a superior threshold switching (TS) behavior with fast switching speed, low OFF-conductance, and large working compliance current^296^ (Figure 15a, 15f, and 15k). A bilayer structure GeTe/Al_2_O_3_ facilitates the ions to form a thicker filament at the interface of GeTe and Al_2_O_3_ due to their different ion dynamics, and by adjusting the thickness of Al_2_O_3_, the device can be tuned between a controllable memory and a TS device.^92^ By adding a TiO_*x*_ buffer layer to a SiO_*x*_ switching layer, the filament becomes cone-shaped, which makes it hard to dissolve spontaneously, tuning the device behavior from volatile to nonvolatile.^267^ A HfO_2_/Ta_2_O_5_ structure facilitates thin filaments at their interface with an easier rupture.^224^ An ALD-HfO_*x*_/RF-HfO_*y*_/ALD-HfO_*x*_ structure, with the Ag preferring to cluster inside the ALD-HfO_*x*_ layer and move fast inside RF-HfO_*y*_ layer, leads to thin filaments inside the HfO_*y*_ layer for fast rupture and low leakage current, and thick residue inside the HfO_*x*_ layer, acting as filament tips, for high selectivity, high-ON current, fast switching speed and high endurance.^260^ This is likely related to the fact that ALD deposition leads to a denser hafnia layer than RF sputter deposition. A TiN/Cu-GeSe/TiN structure creates Jar-shaped Cu filaments that can move back and forth under voltage bias, inducing a well-shaped threshold switching curve for selector applications with fast switching speed and large endurance.^297^ HfO_*x*_/HfO_*y*_, two layers with different oxygen concentrations, is built to realize Ag filament rupture facilitated by oxygen vacancy migration and achieve asymmetric threshold switching behavior.^298^ The attraction of Ag by the high oxygen content in HfO_*y*_ leads to forming-free devices as well. A combination of ALD-HfO_*x*_/PEALD-HfO_*y*_, with the ALD layer having higher mobility than that of the PEALD layer, achieves cone-shaped filaments that rupture easily within the PEALD layer, leading to better uniformity and endurance of the devices.^299^

A layer with extremely low ion mobility is generally used for two purposes. By inserting it between the active electrode and the switching layer, overinjection of ions from the active electrode side is avoided, and the device after the formation process forms filament residues within the buffer layer, which act as sharp electrodes to enhance the electric fields and confine the filament locations. By inserting it between the inert electrode and the switching material, the overgrowth of a filament is avoided, leading to faster relaxation, better endurance, and asymmetric switching behavior. A TiN layer is used as the diffusion barrier for Ag to avoid the overinjection of the Ag into the switching layer, improving the device endurance and reliability.^44^ SrTiO_3_ doped with Ag limits the silver injection into the SiO_2_ switching layer and improves the device reliability.^226^ TiO_*x*_N_*y*_ acts as a buffer layer to limit silver injection and realizes self-compliance with the threshold-switching device.^302^ TaO_*x*_ is used as a buffer layer to avoid overinjection of Ag into the ZnO layer and achieves low threshold voltage, fast switching, and sharp slope^300^ (Figure 15b, 15g, and 15l). A TiN layer is used to avoid overinjection of Ag into the switching layer to achieve good thermal stability and electrical reliability.^82^ This study also shows that the thickness of the TiN layer needs to be explored carefully for optimized performance. Graphene with defects is used as an injection barrier, facilitating thin filament formation for high ON/OFF ratios and self-compliance,^303^ or achieving threshold switching with a large current compliance by limiting the filament size during switching.^304^ SiO_2_ served as a diffusion barrier layer between CeO and Pt to retard Ag migration and suppress filament overgrowth, resulting in high selectivity, high endurance, low variation, and asymmetric switching behavior^301^ (Figure 15c, 15h, and 15m).

A layer with an extremely high ion mobility may be introduced in contact with the active electrode. In this case, the ions tend to diffuse into this layer spontaneously without any voltage stimulation and form a partial filament, resulting in a forming-free device. Besides, the preformed filament may act as a sharp electrode and enable a better switching performance. AIZS quantum dots are demonstrated to facilitate Ag migration with high mobility and enhanced electric field, and they can guide the Ag to form thin and localized filaments for further migration into the switching layer^117,143,268^ (Figure 15d, 15i, and 15n). MXene also guides the Ag filament formation in a SiO_2_ switching layer.^305^

An insertion layer with doped active ions may act as an ion reservoir during switching, as well as avoid overinjection issues due to its controlled ion concentration, enhancing the switching endurance and uniformity. A SrTiO_3_ layer with doped Ag is used as a reservoir for controlled Ag ion injection^226^ (Figure 15e, 15j, and 15o). SiO_*x*_ doped with Ag can also act as the reservoir of Ag ions.^262^ CuS is used as the reservoir of Cu ions.^263^ A TaO_*y*_ layer with rich oxygen vacancies is used as the reservoir of oxygen vacancies.^51^ A halide perovskite layer is used as the Br ion reservoir.^116^

### 5.4 Electrode Optimization

**Figure 16:**
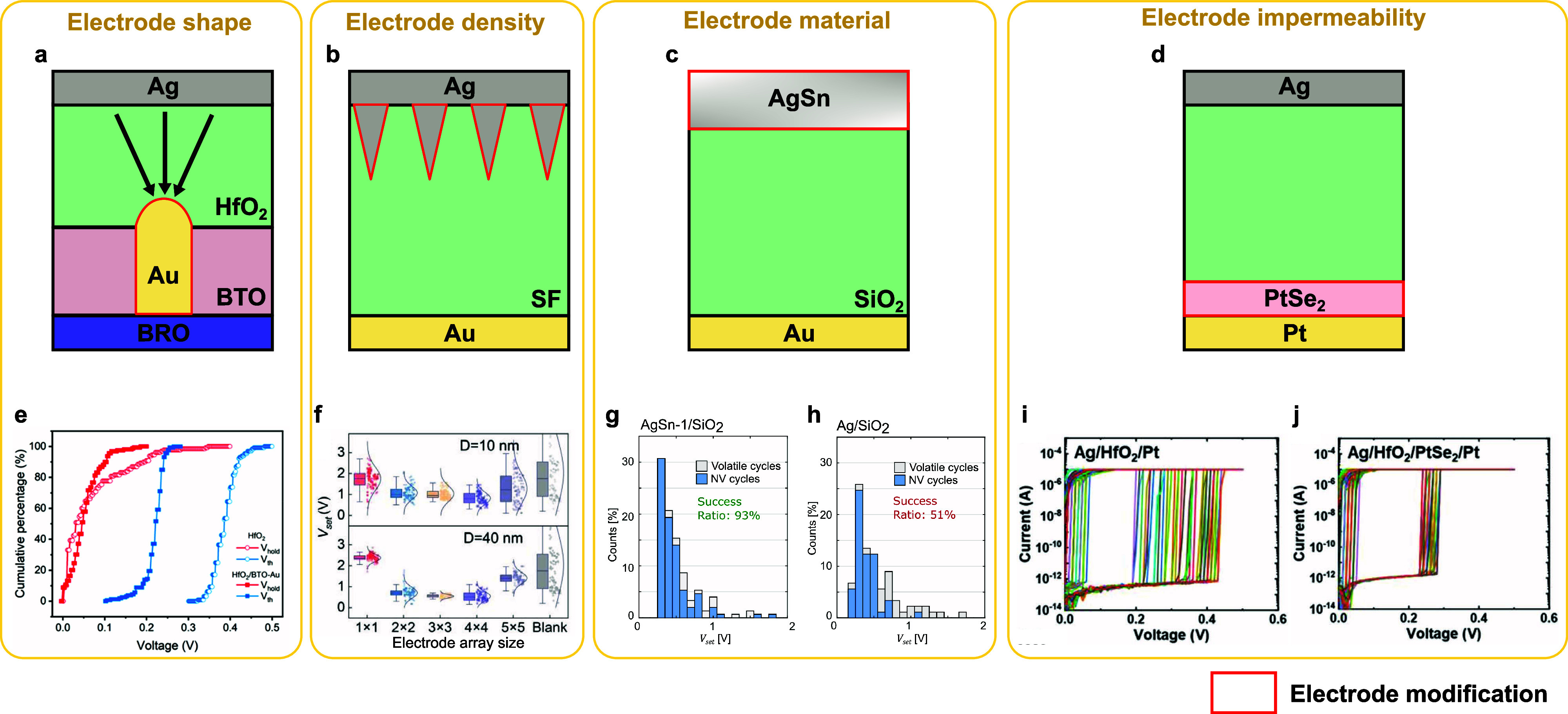
Electrode modifications of IMDs. (a, e) Sharp electrodes concentrate the electric field during switching, leading to better uniformity and lower threshold voltages.^199,306^ (b, f) The tuning of the electrode density results in the change of switching voltages and variations.^142^ (c, g, h) Alloy active electrodes result in better switching uniformity and device yield compared to pure metal electrodes.^290^ (d, i, j) Impermeable inert electrodes avoid the injection of active ions into the electrode, resulting in better device uniformity and endurance.^307^ (e) Reproduced with permission from ref (199). Copyright 2023, American Chemical Society. (f) Reproduced with permission from ref (142). Copyright 2024, Wiley-VCH. (g, h) Reproduced with permission from ref (290). Copyright 2023, American Chemical Society. (i, j) Reproduced with permission from ref (307). Copyright 2023, Wiley-VCH.

The electrode design of the IMDs is an important aspect that needs to be considered to optimize the IMDs. Various methods are explored to make the electrode sharper, which may enhance the local electric field intensity and avoid the overinjection of the active ions, for faster switching speed, lower energy consumption, longer endurance, and better reproducibility. The sharp electrode can be fabricated via special patterning methods. For example, Ag electrode tips are patterned via direct thermal-scanning probe lithography (t-SPL) on silk fibroin^142^ or via t-SPL on polyphthalaldehyde (PPA) and a following reactive ion etching (RIE) through the PPA layer into the SiO_2_ layer.^294^ E-beam lithography (EBL) and thin film deposition are used to create Ag nanodot electrodes with <50 nm diameters.^308^ Ag nanodots or nanoislands are also created by using anodic aluminum oxide (AAO) templates as the electrode sites.^253,309^ Cu electrode cones are fabricated by the isotropic wet-etching of the Cu layer under a photoresist mask.^94^ Inert Au nanorod electrodes are also created via Au adatom nucleation in BaTiO_3_ (BTO)^199^ or by photolithography in indium tin oxide (ITO),^306^ as shown in Figure 16a and 16e.

The electrode density, or the active ion concentration within the electrode, is engineered to tune the switching dynamics. The amount of Ag electrode tips or nanoelectrodes created by the methods mentioned above can be tuned to optimize the switching endurance, randomness, and hysteresis,^142,192,310^ with Figure 16b and 16f as an example. The density of Ag nanoparticles can be controlled during thin-film evaporation, which is used to tune the ON/OFF ratio, voltage threshold, and endurance of the devices.^67,80,131,254^

The impact of electrode materials on the resistive switching characteristics of memristive devices has been extensively studied and is considered crucial in device fabrication.^311^ This importance stems from the potential chemical interplay that occurs at the interface of the electrode and the switching material. Factors such as cation interdiffusion, phase stability, and interfacial reactions play significant roles in determining the device’s behaviors. Understanding and controlling these interactions are essential for optimizing device performance and reliability. By using alloys (AgTe, AgCu, AgSe, and Cu_*x*_Te_*y*_), instead of single elements (Ag and Cu) as the active electrode, volatile memristors with better endurance and thermal stability are demonstrated,^200,259,282,290,299^ with Figure 16c, 16g, and 16h as an example. By using impermeable inert electrodes such as PtSe_2_ and graphene, the injection of active ions into the inert electrode is avoided, leading to better endurance and uniformity of the volatile switching behavior^264,307^ (Figure 16d, 16i, and 16j).

It is imperative to delve deeper into the influence of electrode materials, shapes, and densities on other important aspects of device operation, including retention time, endurance, switching voltages, and ON/OFF ratios, which are essential for optimizing device performance and reliability in practical applications.

### 5.5 Band Engineering

**Figure 17:**
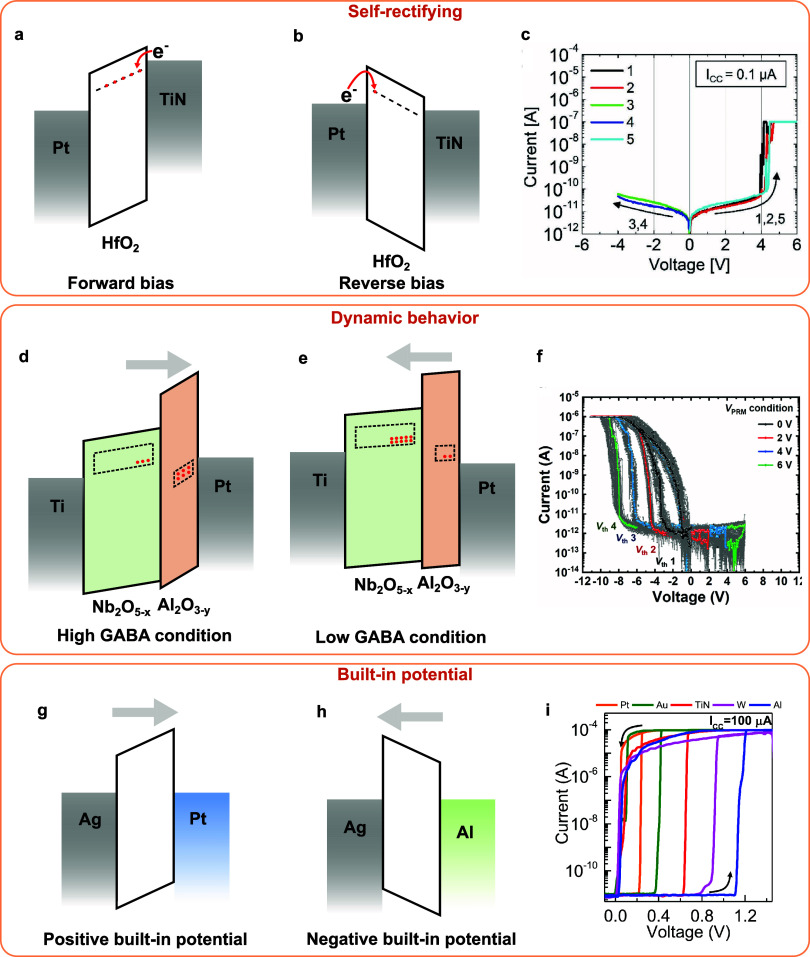
Band diagrams of IMDs can be engineered to introduce special properties. (a–c) A Pt electrode and a HfO_2_ dielectric layer form a Schottky barrier. The barrier (a) allows electron transport when positively biased, and (b) blocks electron transport when negatively biased, leading to (c) asymmetric switching behavior of the device.^265^ (d–f) Nb_2_O_5-x_ and Al_2_O_3-y_ have defect traps at different energy levels to store electrons. A preapplied voltage (V_PRM_) tunes the electron densities trapped in the two layers, resulting in (f) different switching behaviors.^206^ (g–i) Different types of electrode materials have different work functions. (g, h) When paring different electrode materials, the band diagram is tilted and a built-in potential will be introduced, leading to (i) different switching behaviors.^44^ (c) Reproduced with permission from ref (265). Copyright 2018, Wiley-VCH. (f) Reproduced with permission from ref (206). Copyright 2023, Wiley-VCH. (i) Reproduced with permission from ref (44). Copyright 2019, IEEE.

The electrode-switching layer interfaces or switching layer-switching layer interfaces can form junction barriers, which are used for multiple purposes, such as limiting the maximum current, i.e. self-compliance, limiting the reverse current, i.e. self-rectification, and asymmetric switching (Figure 17a to 17c). The interfaces of Pt/TiO_*x*_,^39^ Pd/TiO_*x*_,^101^ Pt/HfO_2_,^265,312^ and Pt/Ta_2_O_5_^313^ form Schottky-like barriers and realize self-rectifying effects to avoid leakage current during reverse biasing. Ag/a-SiN_*x*_/p++-Si also shows self-rectifying behavior originating from the a-SiN_*x*_/p++-Si interface.^314^ Besides the interface junction, the entire band diagram structure may influence the ion and carrier migration and lead to biorealistic dynamics. GABAergic dynamics is realized using a double charge trapping layer with the designed band diagram,^206^ as shown in Figure 17d to 17f. A stack of several dielectric materials can realize switching behavior based on multiple interfaces, which greatly enriches the ion dynamics.^29,47^ Electrode pairs with different Fermi levels can bend the band diagram within the dielectric layer, resulting in a built-in electric field that tunes the threshold voltage^44^ (Figure 17g to 17i). The charge distribution inside the dielectric layer can also lead to a built-in potential, which interacts synergistically with the external bias to show dynamic switching behaviors.^30^

### 5.6 Device Geometry

IMDs can be of various geometries. Each geometry has its unique intrinsic properties, which may lead to special benefits and outperform the other geometries in some aspects.

The two-terminal vertical device is compact for high scalability and stackability. The minimum sizes of these devices are only limited by the sizes of their migration paths and conduction filaments. Thus, with atomic defects as the migration paths, the devices can be scaled down to 100 nm or even sub-10 nm.^41,173,294,315,316^

A planar memristive device can have more than two terminals, which makes it suitable for applications involving multiterminal interactions. For example, the 2D memristive layer based on Li movement can be connected to multiple electrodes, to emulate the heterosynaptic behaviors.^181^ The memristive layer based on an Ag nanowire network with PVP insulating layers can have multiple electrodes, whose voltage inputs interact with each other to determine the filament growth paths within the switching materials. The field distribution inside the network and the competition of filament growth lead to the projection of the input signal to high-dimension, which can be utilized for *in*-*materia* computing and memory of spatiotemporal data sets.^161,162^ A planar Ag_2_Se nanowire network can be used for online learning of a spatiotemporal data set via reservoir computing.^167^ Network-based planar memristive devices also enable the fine-tuning of the device properties, like threshold voltages, ON/OFF ratios, and relaxation speeds by tuning the nanowire densities and lengths.^164,166^ An electrolyte-based planar device can realize structural plasticity by the dynamic growth of the filament inside the electrolyte.^149^ A planar device based on polymer materials with spatially distributed Ag doping and multiple electrodes is used for a multiagent evolutionary network.^132^

Transistor-type memristive devices use the gate dielectric layers to store active ions and the channel materials as conductive layers, avoiding the limited choices of the switching material in two-terminal devices, which need to be both ion and electron conductor, and facilitating more flexible memristive device design. The separation of input gate voltage and output drain-to-source current also leads to better energy efficiency and endurance, and enables the capability to drive the cascaded circuitry.^202^ Besides, transistor-type devices enable multiple input terminals for novel applications. Multiple gate electrodes are added on one transistor-type device to emulate the spatiotemporal integration of multiple synaptic inputs connected to a biological dendrite.^27^ Switching dynamics of a memristive transistor can be tuned via its gate voltage while its drain or other gates are used to receive input signals, enabling the reconfigurability of the device.^28,234^ Inhibitory input and excitatory input are allowed simultaneously via two types of stimuli (e.g., electrical and optical) on the memristive transistor to emulate heterosynaptic behaviors.^317^ The top gate, bottom gate, and drain of a memristive transistor are used to receive different inputs for associative learning.^147,148^ The gate channel of a memristive transistor can be used for chemical sensing or biointerface.^40,45^

## 6 Outlook of Ion-Based Memristive Devices

### 6.1 Challenges

Despite their promising dynamic capabilities, IMDs still face several challenges that hinder their widespread applications. In the previous Section 4, we comprehensively reviewed the requirements for different applications based on IMDs. In general, all applications benefit from devices with better scalability, reproducibility, and energy efficiency. Besides, each application also has some unique requirements to be achieved. For the artificial neuron, the operation speed, the plasticity, and the stochasticity are the key features to be improved. For the artificial synapses, the long-term memory, the short-term memory, and the conductance range are the extra factors that should be considered. For the artificial dendrites, the input density is a unique factor for realizing spatiotemporal integration. For reservoir computing, the device is desired to have a large number of states within its operation range. For selectors, the dynamic range and operation speed are extremely important. For hardware security, the controllable stochasticity of the devices is highly desired. These properties are not only important for the development of biorealistic computing systems using IMDs but also significant for boosting the performance of general computing systems based on IMDs. Within this section, we summarize the needs to improve these factors into six major challenges: scalability, reproducibility, energy efficiency, dynamic range, switching speed, and memory effect. We explore the potential strategies to overcome them for more effective and reliable applications of IMDs.

#### 6.1.1 Scalability

The scalability of the memristive devices is crucial to approach the effective device density of biological neural systems (Figure 12) and is necessary to realize large-scale neuromorphic computing systems.

On the device level, ionic resistive switching based on electrochemical metallization or valence change generally depends on the formation and rupture of localized conduction filaments, thus the minimal device size is limited by the largest dimension of the filaments. Inspired by this property, one potential way to scale the memristive device is to use the single-defect atomristor, which is based on a one-atom-thick switching layer. A threshold-switching device with quantized-conductance single-atom contact has been reported, composed of Ag/hBN/Pt. The switching mechanism is based on monatomic boron defects in the hBN layer, where the switching layer thickness is reduced to a monolayer of hBN(∼0.33 nm) and the effective switching area is scaled down to a single-atom defect.^175^ A type of forming-free nonvolatile atomristor is based on single-layer atomic sheets of transient metal dichalcogenides (TMDs) and the switching mechanism is attributed to ionic diffusion and filament formation with the help of defects in TMD layers, like vacancies and grain boundaries.^318^ Reducing the sizes of the electrodes to the nanoscale can also help increase the device density. The fabrication of a vertical 1D-0D-1D vdW device with an ultrasmall area of 1 nm^2^ has been realized by vertically cross-stacking top carbon nanotubes onto molecularly assembled bottom carbon nanotubes.^319^

On the circuit level, to select individual devices for reading and writing, the traditional 1T1R or 1S1R design is commonly used to overcome the sneak path current issue. However, the addition of transistors imposes more challenges on the downscaling of memristors as well as the upscaling of the array by 3D stacking. The passive crossbar array features a simple geometric structure and the absence of transistors, which is promising for high-density embedding of memristors. One way to solve the sneak path current issue in passive crossbar arrays is to use the self-rectifying memristors, which possess a high nonlinearity during switching and thus need no transistors or selectors. A type of self-rectifying memristors based on Hf_0.8_Si_0.2_O_2_/Al_2_O_3_/Hf_0.5_Si_0.5_O_2_ trilayer exhibited large asymmetry and nonlinearity, and the operation of individual memristors was experimentally proven to be feasible in 320-by-320 passive crossbar arrays.^320^ For those devices that are not self-rectifying, a complementary resistive switching scheme consisting of two antiserial memristive elements can drastically reduce energy consumption and solve the sneak path current issue since all complementary resistive switching exhibits the same high resistance independent of the stored binary data, which makes it possible to fabricate large-scale passive crossbar arrays.^321^

#### 6.1.2 Reproducibility

The reproducibility issue of memristive devices includes the D2D and C2C variations of the devices, which are facing great challenges both in the lab and in industry.^322^

Larger-scale memristor crossbar arrays are crucial for the realization of high-density and high-capacity neuromorphic chips toward their development in next-generation information storage and neuromorphic computing. Achieving wafer-scale uniformity and yield is challenging regarding both material compositions and fabrication methods. Finer crystals and more controllable defects in the resistive switching layer are helpful toward higher yield and better uniformity.^323^ Such demand calls for novel deposition technologies with large-scale thin film uniformity and atomic-level precision. As reported, a memristor crossbar array has been demonstrated using wafer-scale polycrystalline HfSe_2_ grown by molecular beam epitaxy and a metal-assisted van der Waals transfer technique.^324^ High-density and high-yield memristive crossbar arrays can be fabricated using chemical vapor deposition (CVD)-grown hBN as the resistive switching material.^173^ Solution-processed two-dimensional wafer-scale MoS_2_ memristor arrays are also reported to show low device variations as well as excellent endurance, long memory retention, and high analog ON/OFF ratio.^325^

The nonuniformity of single device operation from cycle to cycle could be attributed to ion migration variations over time. The switching variability can be suppressed by one-dimensional channels in epitaxial dielectrics^326^ or confinement nanolayers.^327^ Random telegraph noise is another important factor that leads to the C2C variation and is overcome by an electrical denoising process that can reduce this problem during reading by removing the incomplete channels in memristors.^213^ Endurance failure is a reason that limits the C2C uniformity of devices under continuous operation, which usually originates from different types of structural fatigue. These fatigues include undesired redox reactions with electrodes, overgrowth of filaments, or unwanted diffusion (or loss) of filament atoms.^19^ It has been revealed that a switching material (e.g., TaO_*x*_) with two thermodynamically stable phases, namely a conducting phase (e.g., Ta–O solid solution) and an insulating phase (e.g., Ta_2_O_5_), can have a minimal switching variability and over ten-billion switching cycles.^328^ In subsequent work, 10^10^ cycles have been demonstrated experimentally with TaO_*x*_.^329^ HfO_*x*_ is another example of such material, which has also been extensively used for memristors.

Reproducibility of the device also involves achieving controllable stochastic behaviors for the applications requiring stochastic device behaviors. Several strategies can be applied to balance reproducibility and stochasticity for different application requirements. At the material level, one can optimize the ion type, dielectric material, defect concentration, and interface quality of switching layers to minimize unwanted variations while maintaining desirable stochasticity. At the device level, one can tune layer thickness, utilize nanoscale architectures, or incorporate capping layers to constrain the stochasticity of ion migration and filament formation. At the stimulation level, one can use pulses with tailored voltage or current to regulate device responses.

The stochasticity of ion-based memristive devices can be advantageous for applications like neurons and reservoirs for better network performance. Hardware security applications also benefit from stochasticity that can generate unique and unpredictable responses. For synapses and selectors, stochasticity should generally be reduced as much as possible.

#### 6.1.3 Energy Efficiency

With the development of AI, the most advanced models involve billions of parameters and require extremely intense computation for training and inference. The state-of-the-art AI systems like AlphaZero require thousands of parallel computation units, each with 200-W power consumption. In comparison, the human brain operates at 20 W.^7^ Power efficiency is one of the most critical challenges faced by computing hardware for AI and machine learning, which spurs the interest in neuromorphic computing and associated hardware.

Researchers have sought to use traditional CMOS circuits to realize the biorealistic computing hardware. However, this requires intricate designs of analog and asynchronous circuitry to achieve the functionality required for neuromorphic computing, which usually results in large energy consumption.^330,331^ Utilizing the ion dynamics within the memristive devices to implement the biorealistic computing algorithms, the hardware circuitry can be significantly simplified, which is promising for high energy- and area- efficiency. However, the energy consumption estimated from the current neuromorphic circuits with IMDs is still larger than their biological counterparts (Figure 12), which is due to the unoptimized devices and the inefficient circuit design. The capability to operate in a lower voltage and current range of the IMDs needs to be explored using a measurement setup with a higher sensitivity. Special circuit design schemes are needed to keep the IMDs operating within the low-power range and avoid unnecessary power consumption.

More specifically, the energy efficiency of IMDs can be improved from the aspects of voltage, conductance, dimension, and speed. First, memristive devices with an extremely low switching voltage enable the entire neuromorphic circuit to operate at a low voltage range with low energy consumption. Second, both the OFF and ON conductance of the memristive devices need to be lower than what is available now. This would minimize the unnecessary energy consumption when the circuit is resting, as well as the essential energy consumption during circuit operations. Third, a large portion of the energy is usually consumed by the parasitic components of the device, such as parasitic capacitance or resistance. To minimize such waste, the memristive devices as well as the circuits should be scaled down, which will reduce the parasitic power consumption proportionally. Lastly, pushing the artificial neuromorphic circuits to operate at their speed limitation with the shortest input spikes will reduce redundant energy consumption within each pulse.

#### 6.1.4 Dynamic Range and Number of States

The dynamic range and number of accessible states in IMDs are critical for high-performance neuromorphic computing. A wide dynamic range supports more resistance levels. Combined with fine weight adjustments, more conductance states can be achieved to support high precision in analog computing. However, achieving this is challenging due to limitations such as incomplete ion migration^332^ or filament growth constraints.^68^ Efforts to extend the dynamic range have been focused on material optimization and device engineering. Methods such as doping the RS layer^333,334^ or modifying the top electrode^335^ have shown promise. Scalable devices like low-current HfO_2_/TiO_x_ crossbars with nanoscale dimensions^316^ and tantalum oxide memristors integrated with CMOS circuits^336^ demonstrate 6 orders of magnitude in operating range, including low-current operation below 10 μS.

A large dynamic range is beneficial for obtaining more conductance states with IMDs. However, there are other challenges, including ion transport variation, retention drift, and instability of intermediate states, which limit the number of reliably accessible states. Most reported oxide-based devices achieve 6-bit to 8-bit resolution,^337,338^ with the state-of-the-art reaching 11-bit resolution.^213^ However, it would be extremely hard to achieve the floating-point (32-bit) or double-precision (64-bit) resolution, which is the general case of commercial computers, in a single IMD. From the aspect of single devices, confinement strategies, such as using one-dimensional channels in epitaxial dielectrics^326^ or nanolayers,^327^ help suppress variability to get multiple states. Appropriate electrical operation protocols may also help to increase the number of distinguishable states. For instance, it has been demonstrated that a “denoise” voltage can be used to either complete or annihilate incomplete channels of memristors, leading to thousands of distinct and stable device levels.^213^ Besides, novel circuits and algorithms can be developed to utilize multiple devices to compensate for the weight representation errors and achieve arbitrarily high computation accuracy with IMDs.^339^

#### 6.1.5 Operation Speed

The programming speed of IMDs plays an important role in increasing information processing throughput and computing performance. Compared to electron-based transistors, IMDs that rely on ion migration are generally expected to have relatively slow switching speeds. The time scale of ion migration ranges from picoseconds to seconds and is influenced by a variety of internal and external factors.^58^ Under optimal conditions, resistive switching can occur on subnanosecond time scales.^340^

Achieving fast and efficient device response requires comprehensive optimization of design and operating parameters.^341^ One effective strategy is to reduce the activation energy of ion transport to improve the kinetics of ion transport and accelerate the overall switching process,^26^ at the expense of device retention. Additionally, minimizing the physical size of the device can reduce parasitic effects such as resistance and capacitance delays, which often act as bottlenecks to achieving high-speed performance.^342^ To achieve this, advances in fabrication technology that enable precise control of device dimensions and material properties are critical.

In addition to material and structural improvements, leveraging state-of-the-art analytical tools is useful for understanding and optimizing high-speed dynamics in IMDs. Techniques such as radio frequency (RF) measurement systems provide detailed insights into the time-dependent behaviors of ion migration and resistive switching.^341^ These tools not only enable accurate characterization of switching events at ultrafast time scales but also facilitate the identification of performance-limiting factors and the development of strategies to mitigate them.

#### 6.1.6 Memory Effect

The memory effect of IMD includes both long-term memory and short-term memory. Long retention times of the conductance states are desired for the application like artificial synapses. Within IMDs, this can be achieved by using active ions with high activation energies. The retention time of more than 10 years has been achieved with IMDs.^343^ However, this leads to more energy needed to switch the devices. Besides, another way to achieve a longer retention may be to control the filament growing into a more stable shape, which could be realized by building confined migration paths inside the IMDs or by well-designed stimulation strategies to eliminate unstable portions of the filaments.^213^ Other applications, such as artificial neurons, artificial dendrites, and reservoir computing, do not require stable memory states but instead require memory decay to be controlled within a certain time range. This short-term memory effect can be tuned from a few nanoseconds to a few seconds by using active ions with different interfacial energies. Besides, the combination of different switching materials and electrode materials can be used to engineer the interface structures and band diagram of the devices, as discussed in Section 5, resulting in a better controllability of the ion relaxation behaviors.

### 6.2 Promising Directions

**Figure 18:**
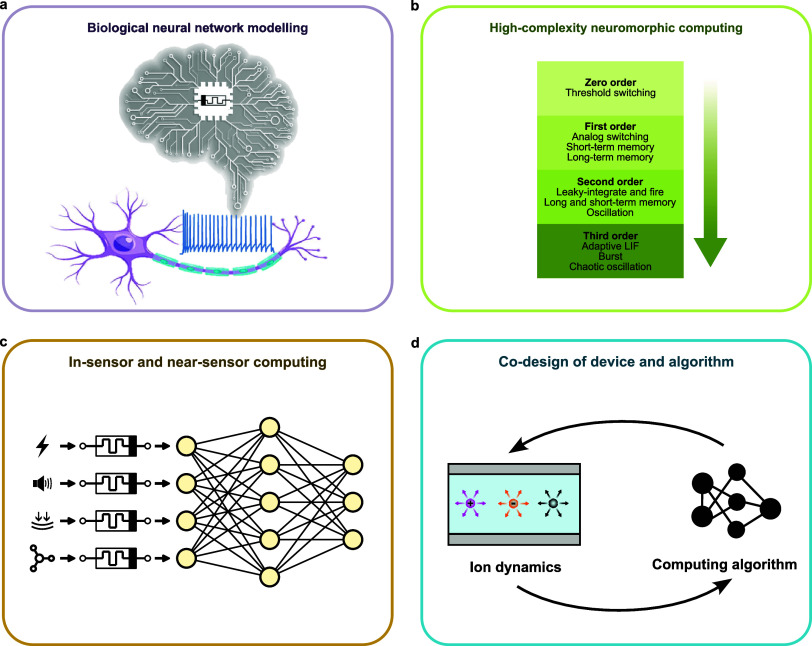
Emerging research directions to realize advanced biorealistic computing systems. (a) The hardware system composed of IMDs can emulate the behaviors of biological neural networks fast, efficiently, and faithfully.^7^ (b) The nonlinear and complex dynamics of the IMDs can realize high-complexity neuromorphic computing systems.^10^ (c) The sensing and encoding capabilities of IMDs can do in-sensor and near-sensor computing.^344^ (d) Co-optimization of the devices and algorithms can be achieved by customizing the device dynamics to reach the requirements of algorithms precisely and customizing the algorithms to utilize the device dynamics thoroughly.^345^

The utilization of the rich ion dynamics within IMDs is still a nascent concept and has not yet been fully explored. Numerous promising perspectives deserve further investigation. In this section, we discuss four emerging directions that are essential for advancing biorealistic computing systems and hold significant potential to fully leverage the dynamic behaviors within IMDs (Figure 18).

#### 6.2.1 Biological Neural Network Modeling

The development of neuromorphic computing is based on the understanding of the human brain. Besides probing the real brain clinically, another important way is to faithfully model the biological neural system operation inside the brain to figure out the embedded learning mechanisms (Figure 18a). Considering the scale and complexity involved in biological systems, hardware emulations are highly desired for speed, efficiency, and fidelity, compared to pure software simulations based on CPU and GPU.^7^ CMOS circuitry has been used to build biological system emulators, such as Neurogrid^13^ and BrainScaleS.^14^ However, the intrinsically different operation principles of CMOS and brain components result in complex and inefficient hardware designs. On the contrary, due to their similar dynamics to biological neurons, IMDs are promising candidates to build efficient and faithful biosystem emulators.

Different types of biological neuron models can be explored for emulation, such as tonically active neurons (TANs)^207^ and phasically active neurons (PANs).^346^ Both tonic and phasic neurons are critical in the central nervous system and they share four fundamental behaviors: all-or-nothing firing, refractory periods, spike frequency adaptation, and spike latency.^347^ Other neural components such as synapses and dendrites lack detailed computational models as the neurons, but their behaviors have been extensively studied quantitatively,^216,231,237,348^ which are good references for building the corresponding emulators.

#### 6.2.2 High-Complexity Neuromorphic Computing

It is of potential interest to ascertain how the unique properties of new materials facilitate complex dynamics, defining various complexities in emerging devices and systems^10,46,349^ (Figure 18b). These inherent complex dynamics at the device level pave the way for innovative computing architectures, such as brain-inspired neuromorphic systems, which offer enhanced energy efficiency and computational capacity. Research has also shown that rich complexity can be beneficial for enabling many intricate neuromorphic behaviors and simplifying the neuromorphic system design.^350,351^ The neuromorphic elements with various complexity based on memristive devices^191,352,353^ are explored. However, these implementations of complex dynamics often require peripheral circuit components. For instance, neuronal oscillations are often generated using distinct components such as parallel capacitors and series resistors, rather than a single memristive component. Action potentials have been realized with one NbO_2_ memristor, one parasitic capacitor, and one series resistor.^52^ A Hodgkin-Huxley-like neuron model was constructed using two volatile VO_2_ memristors with extra electrical components,^354^ which allowed the system to exhibit 23 distinct neuronal functions. A transistor-based memristive system also achieved highly complex dynamics with the combination of multiple transistor circuits, which was capable of producing hyperchaos.^355^ The structural changes during switching in materials like NbO_2_ and VO_2_ involve ion motion within a well-defined short-range (unit cell), making them IMD materials in a broader definition. IMDs, through their electro-physicochemical material processes, naturally exhibit complex biomimetic behaviors, making it possible to achieve high-complexity bioinspired computing systems in a simpler and more efficient way.^47,66^ Analog switching, short-term memory, and long-term memory are all first-order behaviors realized by IMDs that involve only one state variable, which is the total amount of active ions inside an IMD.^41,213^ Long short-term memory is a second-order behavior realized by IMDs requiring two state variables, which are the number of active ions with fast dynamics and the number of active ions with slow dynamics.^28,97^ The active ions in the IMDs can potentially have more than two types of dynamics and be separated into more than two state variables, leading to higher-order behaviors such as burst or chaotic oscillation.

The complexity of the biological neural systems also relies on their dynamic structures. The structures inside the brains, such as neurons and synapses, keep growing, dying, and tuning their behaviors during the lifetime of the creatures, which are essential in biological neural networks for achieving continual and lifelong learning.^356^ The complex dynamics of IMDs make them suitable choices for building reconfigurable devices for such evolutionary learning system. By tuning the current compliance during SET processes between μA-level and mA-level, the halide perovskite memristor based on Ag migration can be reconfigured between volatile and nonvolatile behaviors.^116^ Similarly, the ion-modulated memtransistor based on the LiPON gate dielectric layer can be reconfigured between short-term and long-term dynamics by controlling the Li-ion doping inside the LiPON electrolyte with different stimuli.^28^ Proton distribution within NdNiO_3_ can be tuned among neurons, synapses, and memcapacitors by applying different electric fields.^43^

#### 6.2.3 In-Sensor and Near-Sensor Computing

The number of nodes typically used in sensory networks is growing rapidly, leading to large amounts of redundant data being exchanged between sensory terminals and computing units. Besides, in traditional von Neumann architectures, analog sensory data undergo analog-to-digital conversion (ADC) before being temporarily stored in memory or transmitted to processing units. This results in large amounts of unnecessary data conversion, communication, and processing, leading to high energy consumption, slow throughput, and security issues.

Researchers have become interested in putting the data processing unit as close to the sensor as possible, leading to near-sensor computing, where data is processed by units next to the sensors, and in-sensor computing, where data is processed inside the sensors^153,344,357−359^ (Figure 18c). Near-sensor computing architectures place processing units or accelerators alongside sensors to execute specific computational tasks on the component close to the sensor, minimizing costly data transfers between sensors and processors, which can improve energy efficiency and increase processing speed. In-sensor computing architectures use individual sensory devices with dynamic behaviors to process sensory information directly, eliminating the need for a separate sensor/processor interface and enabling the combination of sensing and computing capabilities. These new applications require substantial breakthroughs in materials, device physics, and computing architectures.^360^ Ion dynamics have been recognized as a promising feature to efficiently enable near-sensing and in-sensing computing. Due to the low-power computing with rich ionic motions in the devices, they have been proven capable of sensing and processing information simultaneously, including pressure signals,^121^ visual signals,^361^ tactile signals,^362^ etc.

The memristive devices that are sensitive to biological signals, such as voltages and chemicals, can be used as biointerfaces to detect and process biological signals for applications such as therapeutic intervention of various diseases, chemical detection of toxicology, and drug delivery studies. The similar dynamics of these devices and biological neurons enable them to detect and process the biosignals at the same time. Thus, these devices are promising as brain-machine interfaces with biological neural networks, realizing the cooperation and interaction between the human brain and artificial intelligence. For example, the memristive devices with biovoltage at sub-100 mV range have been developed and utilized for biosignal processing within a bioelectronic interface.^137^ The organic artificial spiking neurons based on organic transistors are implemented in contact with biological neural cells. The biological membrane status influences the ion concentration near the transistors and changes the firing behaviors of the spiking neurons, demonstrating a biohybrid neuron to detect toxin-induced disruptions.^45^ The biorealistic organic electrochemical neuron based on organic transistors can be modulated by secondary ions, such as Na^+^ and Cl^–^, and by biomolecules, such as GABA and glutamine, which are combined with neuron electrodes to demonstrate feedback nerve actuation.^40^

#### 6.2.4 Co-design of Device and Algorithm

Although memristive devices exhibit rich ion dynamics that bear remarkable similarities to biological synapses, dendrites, and neurons, making them promising candidates for future AI and neuromorphic computing, these dynamics alone do not guarantee efficient computation.^64,191,232,345,363^ Successful utilization requires careful co-design and co-optimization of devices, circuits, and algorithms (Figure 18d). For instance, previous literature reports large C2C and D2D variances within the IMDs, which impede their direct applications in most of the current AI systems requiring high-precision calculations.^71,345^ Therefore, co-design and co-optimization have become key focal points in building neuromorphic systems with these devices. There are two major aspects to proceed with the co-design scheme: 1) investigating the potential of leveraging the properties of memristors in traditional machine learning algorithms; 2) exploring new neuromorphic computing algorithms to better utilize the properties of memristive devices.

The IMDs have been used to implement many traditional applications and algorithms. For example, a stochastic number generator is implemented by exploiting the stochastic delay time of switching behavior in memristors, exhibiting evident advantages in scalability, circuit complexity, and power consumption.^67^ The threshold switching probability of the memristive devices could be controlled by applied bias, enabling the implementation of dropout neurons commonly used to mitigate overfitting problems during training.^60^ Memristor-based reservoirs leverage the volatile and stochastic properties of memristors to realize reservoir computing.^135,313,364,365^ The stochastic switching properties of IMDs can simulate random connections in biological reservoirs and extract temporal features from the input signals. D2D variances could be beneficial to certain learning algorithms, where each device focuses on temporal features within different frequency ranges.^55^ However, the benefits of C2C and D2D variations will diminish and be outweighed by drawbacks if the variations are too large. Thus, future improvements are still needed from both device and algorithm aspects to overcome these issues.

Designing specific circuits, algorithms, and systems holistically that fully exploit the intricate dynamics of IMDs is another co-design approach. Many efforts have utilized threshold-switching devices to build LIF neurons for SNN applications.^89,143,192,194^ However, these LIF neurons fail to fully exploit the rich dynamics within the IMDs. Research is in progress to design more comprehensive artificial neurons encompassing more biological features by carefully utilizing the assembly and relaxation of ion channels within the diffusive memristor. Nevertheless, these spiking neurons still show excessively large stochasticity, which is undesired in many SNN algorithms. In comparison, human brains operate with a relatively large noise and have the capability to learn with low precision and sparse spikes, diverging from the high precision requirements of modern deep learning algorithms. It is widely believed that the human brain mitigates this challenge through special spike-frequency and -population-encoding schemes.^366−368^ This inspires the development of SNN systems based on the biorealistic spike-frequency and spike-population encoding schemes, which demonstrate superior robustness and error tolerance to noises, showcasing their potential to enhance network reliability.^366−368^ It is proposed that this enhancement is because neural coding can effectively project temporal information into the spatial domain, thus improving the linear separability of the patterns.^367^ Nevertheless, compared to spike-timing coding, spike-frequency coding may prolong training and inference time and spike-population coding necessitate a larger neuron population. Therefore, the development of brain-inspired algorithms more suitable for IMDs remains an ongoing endeavor and needs future explorations.

### 6.3 Summary

Computation hardware has long been dominated by devices based on the manipulation and transportation of electrons. The concept of leveraging ion dynamics to realize computational functions is still at its nascent stage. However, over the past two decades, the development of various ion-based memristive devices and extensive studies of their mechanisms and applications have begun to reveal the great potential of these devices. The capabilities of IMDs to closely resemble the biological operation mechanisms and emulate biological neuron functions started to be uncovered and valued. Their versatility, stemming from a wide range of material choices, diverse ion switching properties, and flexibility in device configuration design, positions IMDs as a promising candidate to realize biorealistic computing schemes. Despite this promise, current IMDs have not yet met the requirement for building scalable computing platforms or large-scale systems. Key challenges such as scalability, reproducibility, and energy efficiency must be addressed to enable reliable, system-level implementation at the next stage. This calls for further efforts to deepen our understanding of ion dynamics in different materials and to develop precise control strategies for these processes. Moreover, the development of peripheral circuits to support analog and dynamic operations, computing architectures to integrate IMDs, and software algorithms to effectively harness ion dynamics is equally critical to the realization of ion-based computing systems. Biorealistic computing based on ion dynamics, with its fundamentally different approach compared to traditional electronic devices, is not intended to replace existing computing hardware. Instead, it opens new avenues to rethinking computational strategies and dealing with applications that are less suited for traditional computing hardware, such as high-complexity neuromorphic computing and in-sensor or near-sensor computing, which will ultimately expand the horizons of computational hardware, paving the way for artificial intelligence systems to approach or even exceed biological intelligence.
